# Recognition of *Aspergillus fumigatus* Hyphae by Human Plasmacytoid Dendritic Cells Is Mediated by Dectin-2 and Results in Formation of Extracellular Traps

**DOI:** 10.1371/journal.ppat.1004643

**Published:** 2015-02-06

**Authors:** Flávio V. Loures, Marc Röhm, Chrono K. Lee, Evelyn Santos, Jennifer P. Wang, Charles A. Specht, Vera L. G. Calich, Constantin F. Urban, Stuart M. Levitz

**Affiliations:** 1 Department of Medicine, University of Massachusetts Medical School, Worcester, Massachusetts, United States of America; 2 Department of Immunology, Institute of Biomedical Sciences, São Paulo University, São Paulo, Brazil; 3 Umeå Centre for Microbial Research, Laboratory for Molecular Infection Medicine Sweden (MIMS), Department of Clinical Microbiology, Umeå University, Umeå, Sweden; Geisel School of Medicine at Dartmouth, UNITED STATES

## Abstract

Plasmacytoid dendritic cells (pDCs) were initially considered as critical for innate immunity to viruses. However, our group has shown that pDCs bind to and inhibit the growth of *Aspergillus fumigatus* hyphae and that depletion of pDCs renders mice hypersusceptible to experimental aspergillosis. In this study, we examined pDC receptors contributing to hyphal recognition and downstream events in pDCs stimulated by *A. fumigatus* hyphae. Our data show that Dectin-2, but not Dectin-1, participates in *A. fumigatus* hyphal recognition, TNF-α and IFN-α release, and antifungal activity. Moreover, Dectin-2 acts in cooperation with the FcRγ chain to trigger signaling responses. In addition, using confocal and electron microscopy we demonstrated that the interaction between pDCs and *A. fumigatus* induced the formation of pDC extracellular traps (pETs) containing DNA and citrullinated histone H3. These structures closely resembled those of neutrophil extracellular traps (NETs). The microarray analysis of the pDC transcriptome upon *A. fumigatus* infection also demonstrated up-regulated expression of genes associated with apoptosis as well as type I interferon-induced genes. Thus, human pDCs directly recognize *A. fumigatus* hyphae via Dectin-2; this interaction results in cytokine release and antifungal activity. Moreover, hyphal stimulation of pDCs triggers a distinct pattern of pDC gene expression and leads to pET formation.

## Author Summary

While plasmacytoid dendritic cells (pDCs) are known to be important immune cells involved in protection from viruses and tumors, their role in protection against fungal infections is less clear. Our laboratory has been studying the interplay between pDCs and the fungal pathogen, *Aspergillus fumigatus*. Our previous work demonstrated that human pDCs bind to and inhibit the growth of *A*. *fumigatus* hyphae. Moreover, depletion of pDCs rendered mice very susceptible to experimental infection with *A*. *fumigatus*. Here, we show that Dectin-2, a receptor on pDCs, recognizes *A*. *fumigatus* hyphae and contributes to cytokine release and antifungal activity. In addition, using confocal and electron microscopy, we demonstrate that upon contact with hyphae, some human pDCs die and form antimicrobial structures called extracellular traps. Finally, using microarrays, we analyzed human pDC gene expression upon *A*. *fumigatus* infection and found distinct patterns including the activation of genes previously associated with viral infections and apoptosis. These results provide new insights into the mechanisms by which pDCs might help the immune system when confronted with a fungal invader.

## Introduction

*Aspergillus fumigatus* is an opportunistic fungal pathogen with a worldwide distribution. Exposure typically occurs when airborne spores (conidia) are inhaled into the lungs. If the conidia are not contained, they may swell and germinate into hyphae. Invasive aspergillosis (IA) is seen predominantly in immunocompromised patients and is characterized by hyphal invasion associated with tissue destruction [1]. The relatively weak fungicidal activity of the available therapeutic options contributes to the high mortality rates seen in patients with IA [2]. Other clinical manifestations of aspergillosis result from allergic responses to the fungus. Innate immune responses of phagocytes, particularly neutrophils, are essential for effective host defenses against *A. fumigatus*. Toll-like receptors (TLRs) and C-type lectin receptors (CLRs) on phagocytes recognize surface ligands on *A. fumigatus* [3], [4], [5], [6]. Although hyphae grow too large to be phagocytosed, phagocytes spread over the hyphal surface and antifungal activity proceeds via both oxidative and non-oxidative mechanisms. Moreover, dying neutrophils can release DNA and antimicrobial proteins, including calprotectin, as extracellular traps (ETs), which are able to trap hyphal elements [7]. Thus, larger fungal morphotypes, including tissue-invading Aspergillus hyphae, can still be controlled [8]. Macrophages, eosinophils, and mast cells also release ETs [7], [9] although it is unknown whether these cell types can form ETs in response to Aspergillus.

Plasmacytoid DCs (pDCs) rapidly produce copious amounts of type I interferon (IFN) upon stimulation with viruses [10]. In humans, pDCs comprise 0.2%–0.8% of the total peripheral blood mononuclear cells (PBMCs) and express the endosomal Toll-like receptors (TLRs) 7 and 9, but not TLR2, TLR3 or TLR4 any of the cell surface TLRs. Activated pDCs link innate to adaptive immunity by secreting cytokines such as IFN-α and tumor necrosis factor (TNF-α) and by differentiating into mature pDCs with upregulated MHC and costimulatory molecules capable of priming naive T cells [11].

pDCs are widely described to have roles in viral defenses, tumor immunity, autoimmunity, allergy and some bacterial infections [12], [13], [14], [15], [16], [17]. Our group recently described that pDCs detect and respond to *A*. *fumigatus*. We demonstrated that unmethylated CpG-rich motifs in *A*. *fumigatus* DNA stimulate human pDCs to produce IFN-α [18]. In addition, when incubated with hyphae, human pDCs directly inhibit fungal growth via a mechanism that involves *A. fumigatus*-induced pDC death and the release of antifungal mediators including calprotectin. Moreover, following stimulation with *A. fumigatus* hyphae, pDCs release IFN-α and TNF-α via a mechanism that appears to be TLR-independent. Importantly, depletion of pDCs renders mice hypersusceptible to pulmonary and intravenous challenge with *A*. *fumigatus* [19]. In another model of fungal infection, mice resistant to pulmonary paracoccidioidomycosis expanded a subpopulation of pDC that secreted TNF-α, TGF- β and IL-6. This resulted in expansion of interferon-γ-, IL-4-, and IL-17-positive effector T cells [20].

In the present study, we further investigated the interaction between human pDCs and *A*. *fumigatus* hyphae. As fungal recognition appears to be TLR-independent, we investigated the possible involvement of two C-type lectin receptors, Dectin-1 and Dectin-2, which have been demonstrated to bind to *A*. *fumigatus* hyphae [5], [6], [21], [22], [23]. Moreover, as *A*. *fumigatus* induces pDC death, we examined whether pDC ETs (pETs) formed following incubation with hyphae. Finally, to gain further insight into the nature of the pDC response to *A*. *fumigatus* hyphae, we took an unbiased systems biology approach by profiling pDC gene expression following hyphal challenge. We found that human pDCs directly recognize *A*. *fumigatus* hyphae via Dectin-2; this interaction triggers antifungal activity and cytokine release. Following incubation with hyphae, pDCs formed ETs containing citrullinated histone H3. In addition, *A*. *fumigatus* stimulation elicited a distinct pattern of pDC gene expression including up-regulation of genes involved in cell activation, cell migration, cytokine and chemokine production, apoptosis and other biological processes.

## Results

### *A*. *fumigatus* hyphae are recognized by Dectin-2 on pDCs

**Fig 1:**
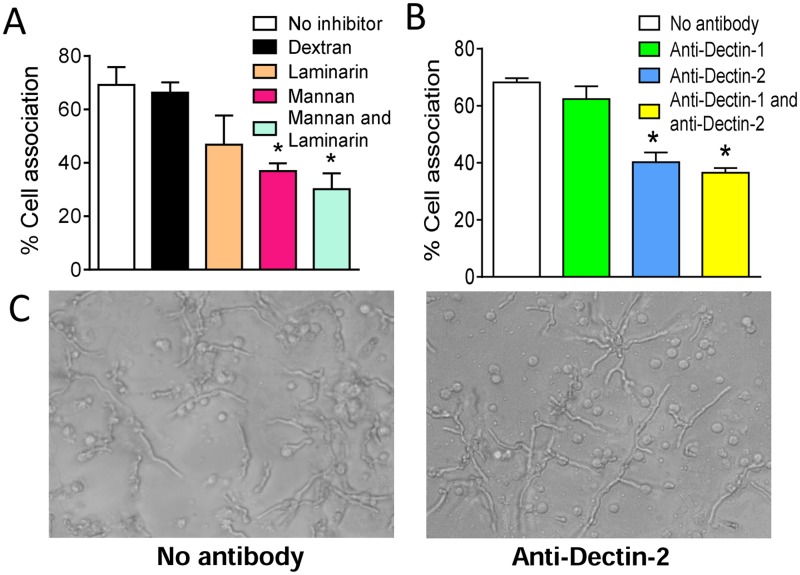
*A*. *fumigatus* hyphae are recognized by the mannose receptor, Dectin-2, on pDCs. Human pDCs were purified from PBMCs fractions using CD304-coated magnetic beads. (A) pDCs were treated with no inhibitors, dextran (1 mg/mL), mannan (1 mg/mL), laminarin (0.5 mg/mL) or mannan and laminarin for 30 minutes. The pDCs (5×10^4^) were then incubated for 2 hr with *A. fumigatus* hyphae (5×10^4^). The cell association was quantified by counting number of pDCs associated (touching or spreading over) with hyphae in 10 different fields. The percent cell association was calculated by dividing the number of pDC associated with hyphae by the total number of pDC counted and then multiplying by 100. (B) Same as in A except pDCs were treated with anti-Dectin-1 (100 μg/mL) and anti-Dectin-2 (100 μg/mL) antibodies for 30 minutes prior to incubation with hyphae. Data represent means ± SE of % cell association from three donors. *P<0.05. (C) Representative photomicrographs of pDCs incubated with *A*. *fumigatus* hyphae under the conditions described in panel B.

Initial experiments focused on determining which PRRs contributed to the recognition of the hyphal morphotype of *A. fumigatus by* human blood pDCs. pDCs were incubated with *A. fumigatus* hyphae for 2 hr at 37°C in the presence of mannans (which blocks mannose receptors) and/or laminarin (which blocks β-1,3-D-glucan receptors). Control wells contained no added polysaccharides or the α-glucan, dextran. pDCs treated with mannans inhibited the association between pDCs and *A fumigatus* hyphae by greater than 50% (Fig. 1A). In contrast, laminarin or dextran treatment did not significantly alter binding of pDCs to the hyphae although there was a trend towards less binding in the presence of laminarin. There was also a trend towards reduced binding when comparing the combination of laminarin and mannan with mannan alone. The above results suggest that mannose receptors are largely (albeit not solely) responsible for the recognition of *A*. *fumigatus* hyphae by pDCs. As human pDCs reportedly express the mannose receptor Dectin-2 but not the β-glucan receptor Dectin-1 [24], [25], we hypothesized that Dectin-2 is a major pDC receptor for *A*. *fumigatus* hyphae. Indeed, blocking antibodies directed against Dectin-2 significantly decreased the number of pDCs found in association with hyphae (Fig. 1B). In contrast, blocking antibodies directed against Dectin-1 were not inhibitory. Representative photomicrographs of pDCs incubated with *A*. *fumigatus* hyphae in the presence or absence of blocking antibodies to Dectin-2 are shown in Fig. 1C.

### Dectin-2 is involved in antifungal activity and cytokine release by pDCs stimulated with *A*. *fumigatus* hyphae

**Fig 2:**
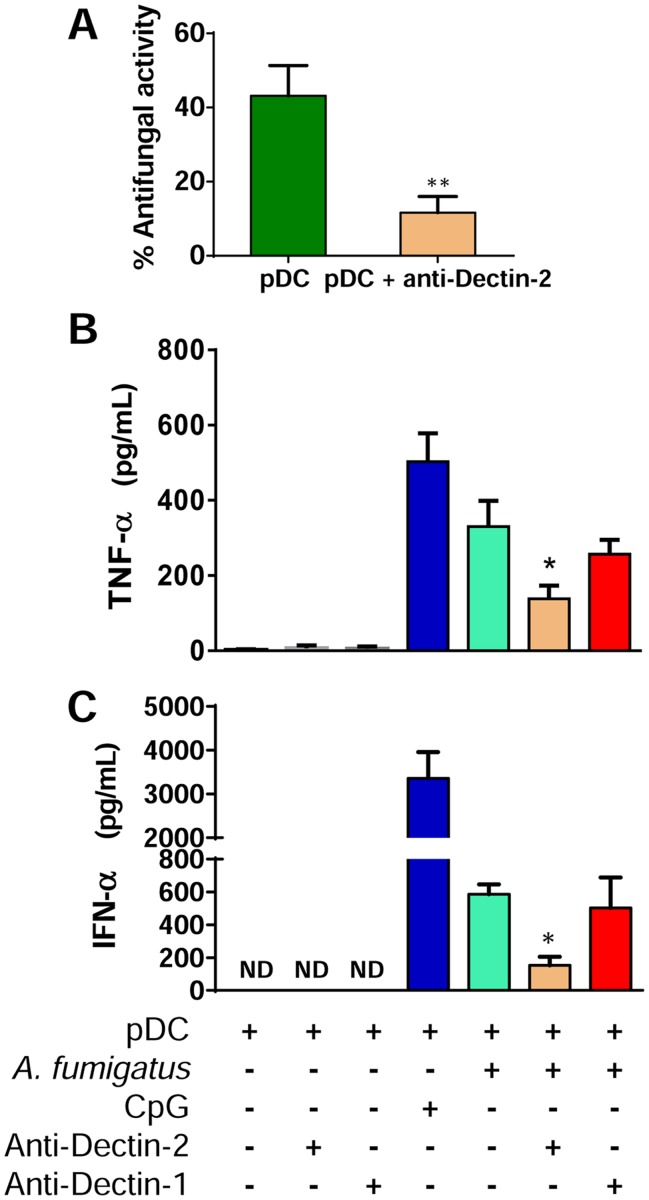
Dectin-2 is involved in antifungal activity and cytokine release by pDCs stimulated with *A. fumigatus* hyphae. (A) Human pDCs were isolated from PBMCs using magnetic beads. The pDCs (5 × 10^4^) were incubated with *A. fumigatus* hyphae (5 × 10^3^) for 2 hr in the presence or absence of anti-Dectin-2 antibody. Antifungal activity of pDCs was then measured by the XTT assay. Data represent means ± SE from three donors, each tested in triplicate. *P = 0.004. (B-C) *A. fumigatus* conidia (5 × 10^4^) were plated in 96-well plates and grown in pDC media to hyphae. pDCs (5 × 10^4^) were left untreated or incubated with anti-Dectin-2 (100 μg/mL) or anti-Dectin-1 (100 μg/mL) antibodies and then added to the hyphae. Control wells contained pDCs only, pDCs and antibodies, or pDC and CpG (10 μg/mL). After 6 hr, the supernatants were removed and analyzed by ELISA for TNF-α (B) and IFN-α (C). Data represent means ± SE of cytokine concentrations from two (IFN-α) or three (TNF-α) pDC donors, each tested in triplicate. *P<0.05 comparing cytokine secretion by *A*. *fumigatus*-stimulated pDCs with *A*. *fumigatus*-stimulated pDCs treated with anti-Dectin-2 antibody.

To assess the contribution of Dectin-2 to pDC-mediated antimicrobial activity, pDCs were incubated with *A. fumigatus* hyphae for 2 hr at 37°C in the presence or absence of blocking anti-Dectin-2 antibody. Antifungal activity was measured by the XTT assay. We found that blocking Dectin-2 resulted in a profound reduction in antifungal activity against *A. fumigatus* hyphae (Fig. 2A). Next, we examined whether Dectin-2 recognition of *A. fumigatus* hyphae could impact immune responses by triggering cytokine release. pDCs were stimulated with *A. fumigatus* hyphae for 6 hr at 37°C in the presence of anti-Dectin-2 or anti-Dectin-1 blocking antibodies. As negative and positive controls, pDCs were left unstimulated or stimulated with the TLR9 ligand CpG. Concentrations of TNF-α (Fig. 2B) and IFN-α (Fig. 2C) were measured in the supernatants. We found that release of TNF-α and IFN-α was reduced when the pDCs were blocked with anti-Dectin-2 but not with anti-Dectin-1 antibody.

### *A*. *fumigatus* hyphae trigger signaling responses by Dectin-2 and FcRγ cooperation

Transfected B3Z cells were utilized to further examine the role of Dectin-2 in hyphal recognition. Dectin-2 can couple to the Syk-CARD9 innate signaling pathway to activate DCs and regulate adaptive immune responses to fungal infection. Unlike Dectin-1, Dectin-2 couples to Syk indirectly, through association with the FcRγ chain [26]. To assess the ability of Dectin-2 to recognize *A*. *fumigatus* and trigger cell activation, we used B3Z cells containing a reporter plasmid for NFAT coupled to the β-galactosidase gene. These cells were also transduced with Dectin-2 alone, Dectin-2 and FcRγ, FcRγ alone or Dectin-2 and a signaling-deficient mutant FcRγ chain. The four cell lines were then stimulated with zymosan (a ligand for Dectin-2) [26], *A*. *fumigatus* conidia or *A*. *fumigatus* hyphae.

**Fig 3:**
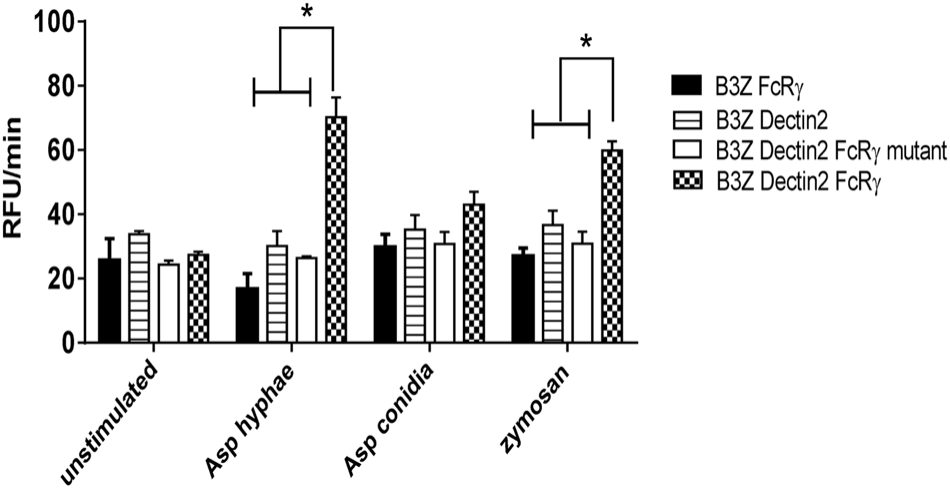
*A*. *fumigatus* hyphae trigger signaling responses by Dectin-2 and FcRγ cooperation. B3Z cells (2 × 10^5^) containing a reporter plasmid for NFAT coupled to the β-galactosidase gene were transduced with WT FcRγ chain, Dectin-2, Dectin-2 and a signaling-deficient mutant of FcRγ chain Dectin-2 and WT FcRγ chain. Cells were either left unstimulated or stimulated with *A*. *fumigatus* hyphae (1 × 10^5^), *A*. *fumigatus* conidia (1 × 10^5^) or zymosan (100 μg/ml). Fluorescence intensity, a reflection of NFAT activity, was measured at 5 min intervals for 1 hr. Data are means of RFU/min ± SD of duplicate wells and are representative of two independent experiments. * *P*<0.05.

Following 2 hr of hyphal or zymosan stimulation, a significant increase in NFAT reporter activity was seen in B3Z cells that were co-transduced with Dectin-2 and FcRγ chain (Fig. 3). The other B3Z cell lines, including the line expressing the mutant FcRγ chain and Dectin-2, did not increase their signal in response to either *A*. *fumigatus* hyphae or zymosan, as determined by NFAT reporter activity. Conidia did not stimulate significant increases in β-galactosidase activity in any of the cell lines tested. In two independent experiments, each performed in duplicate, *A*. *fumigatus* hyphae did not stimulate detectable β-galactosidase activity in the absence of cell lines.

### Formation of pDC extracellular traps after incubation of pDCs with *A*. *fumigatus* hyphae

**Fig 4:**
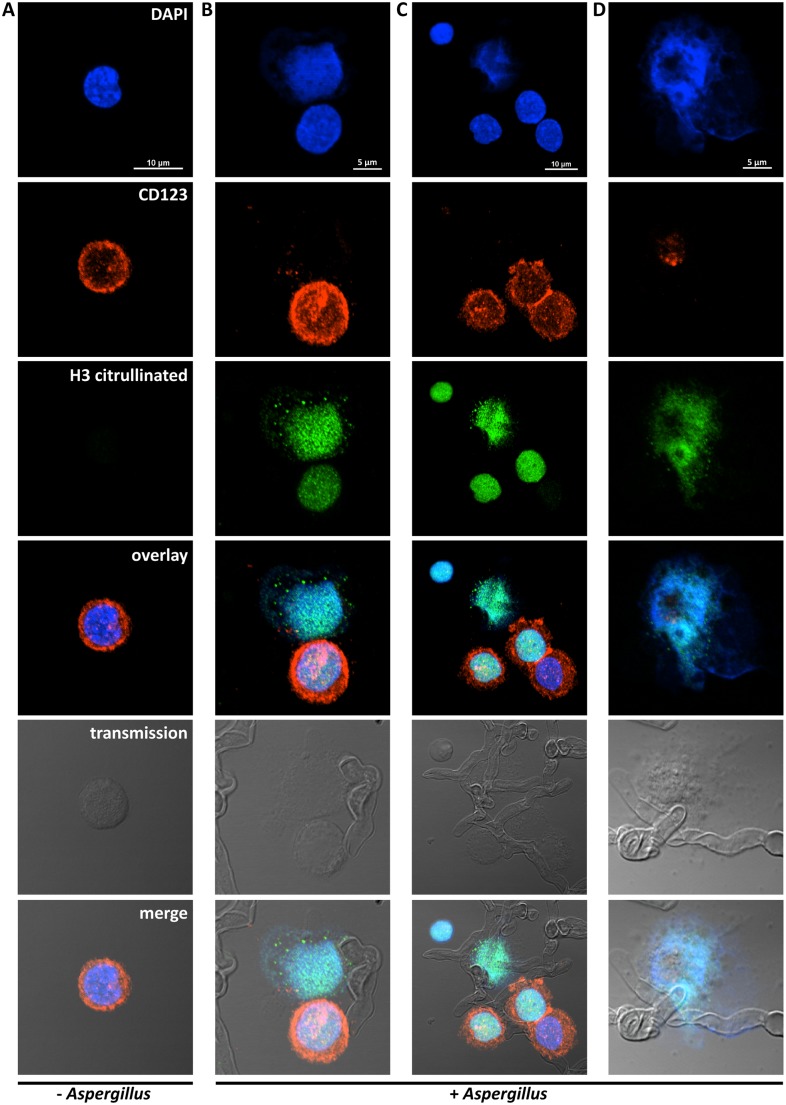
Detection of pDC extracellular traps (pETs) by immunofluorescence. Human pDCs (2 × 10^5^/well) were left unstimulated (Unstim) or stimulated for 4 hr with *A. fumigatus* hyphae (2 × 10^5^). pDCs associated with *A. fumigatus* hyphae showed evidence of ETosis. pETs were visualized by indirect immunofluorescence using primary antibodies against citrullinated histone H3 and the pDC marker CD123. Alexa Fluor 488- and 568-conjugated secondary antibodies were used for visualization of citrullinated histone H3 (green channel) and CD123 (red channel), respectively. DNA was stained with DAPI. Images were captured with a confocal microscope and a 60x oil immersion objective. Wavelengths of 405 nm (diode), 488 nm (Argon), and 543 nm (HeNe) were used to excite DAPI, Alexa Fluor 488 (and transmission images), and Alexa Fluor 568, respectively. Images were captured in separate passes to avoid cross talk and are presented as maximum intensity projections from Z-stacks. (A) Unstimulated pDCs. (B-D) Aspergillus-stimulated pDCs demonstrating pETs formation. The data are representative of three independent experiments.

**Fig 5:**
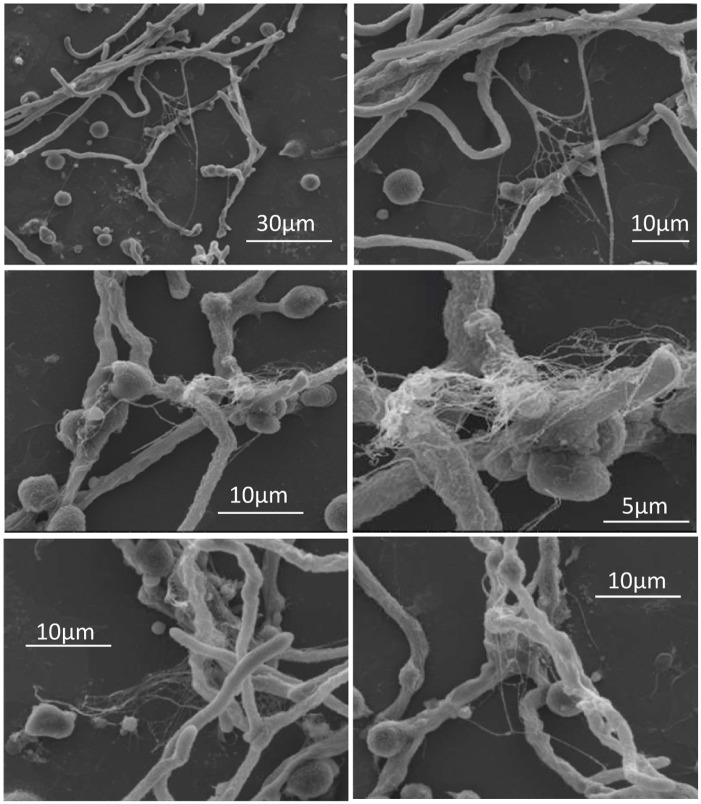
Scanning electron microscopy of pDCs incubated with *A*. *fumigatus* hyphae. Human pDCs (2 × 10^5^/well) were stimulated for 4 hr with *A. fumigatus* hyphae (4 × 10^5^). The samples were then fixed and imaged by scanning electron microscopy. Areas with pETs are shown at low (left panels) and high (right panels) magnification. The data are representative of two independent experiments.

It was recently reported that neutrophils sense microbe size and selectively release neutrophil extracellular traps (NETs) in response to large pathogens such as *C*. *albicans* hyphae and extracellular aggregates of *Mycobacterium bovis* [27]. In addition, it was demonstrated that netting neutrophils are major inducers of type I IFN production [28]. This, along with our demonstration that pDC produced IFN-α after binding *A*. *fumigatus* hyphae (Fig. 2C and [19]), led us to ask whether pDCs can make extracellular traps following contact with *A*. *fumigatus* hyphae. Two complementary techniques, confocal microscopy and scanning electron microscopy (SEM), were used to determine whether extracellular traps are formed by pDC following incubation with *A. fumigatus* hyphae. For the confocal studies, following a 4 or 6 hr incubation of pDCs with hyphae, the samples were stained for DNA, the pDC specific receptor CD123, and citrullinated histone H3. Unstimulated pDCs had intact nuclear DNA as measured by DAPI staining, labeled brightly with anti-CD123 antibody, and had no detectable staining with antibodies directed at citrullinated histone H3 (Fig. 4A). In contrast, following incubation with *A*. *fumigatus*, pDCs that were associated with hyphae exposed disrupted, extracellular DNA that co-localized with citrullinated histone H3 (Fig. 4B-D). When the interactions of pDCs with hyphae were examined by SEM, areas colonized by *A. fumigatus* showed ET-like structures spread over fungal surfaces (Fig. 5). Following incubation with *A*. *fumigatus* hyphae, ET formation was observed in approximately 1% of the pDCs. More precise quantification proved to be problematic; some ETs appeared to be in the process of being formed and for well-formed ETs, it could be difficult to tell whether the ET was from one or more pDCs. The appearance of the observed structures is very similar to that described for NETs [29], [30], [31]. Taken together, these observations strongly suggest that pDCs are able to make ETs upon *A*. *fumigatus* infection *in vitro* and we propose the term pETs (pDC extracellular traps) for these structures.

### The transcriptome of pDCs following stimulation with *A. fumigatus*

Activated pDCs link innate to adaptive immunity by secreting cytokines such as IFN-α and TNF-α and by differentiating into mature pDCs with up-regulated MHC and costimulatory molecules capable of priming naive T cells [11]. To begin to better understand the full role of pDCs in defense against fungal infections, we took an unbiased approach by determining the human pDC transcriptome upon challenge with *A*. *fumigatus* hyphae. The spectrum of changes in gene expression was examined in pDCs from three blood donors at 2 and 4 hr following incubation with hyphae. Comparative controls included unstimulated and pDCs at 4 hr following stimulation with CpG.

Discriminant microarray analysis demonstrated significant changes in the pDCs transcriptome after 2 and 4 hr of interaction with *A*. *fumigatus* hyphae. Of the 53,617 gene probe sets represented on the expression array, we identified a total of 2,309 up-regulated and 1,638 down-regulated genes for pDCs from at least one donor. When we looked for concordant expression for pDCs from all three donors, statistical analyses found 79 regulated genes (44 up-regulated and 35 down-regulated) after 2 hr and 250 regulated genes (179 up-regulated and 71 down-regulated) after 4 hr of pDC-Aspergillus hyphae interaction (S1 Table). Of the 44 genes up-regulated at 2 hr, 12 continued to be up-regulated at 4 hr; of the 35 genes down-regulated at 2 hr, 10 continued to be down-regulated at 4 hr.

**Fig 6:**
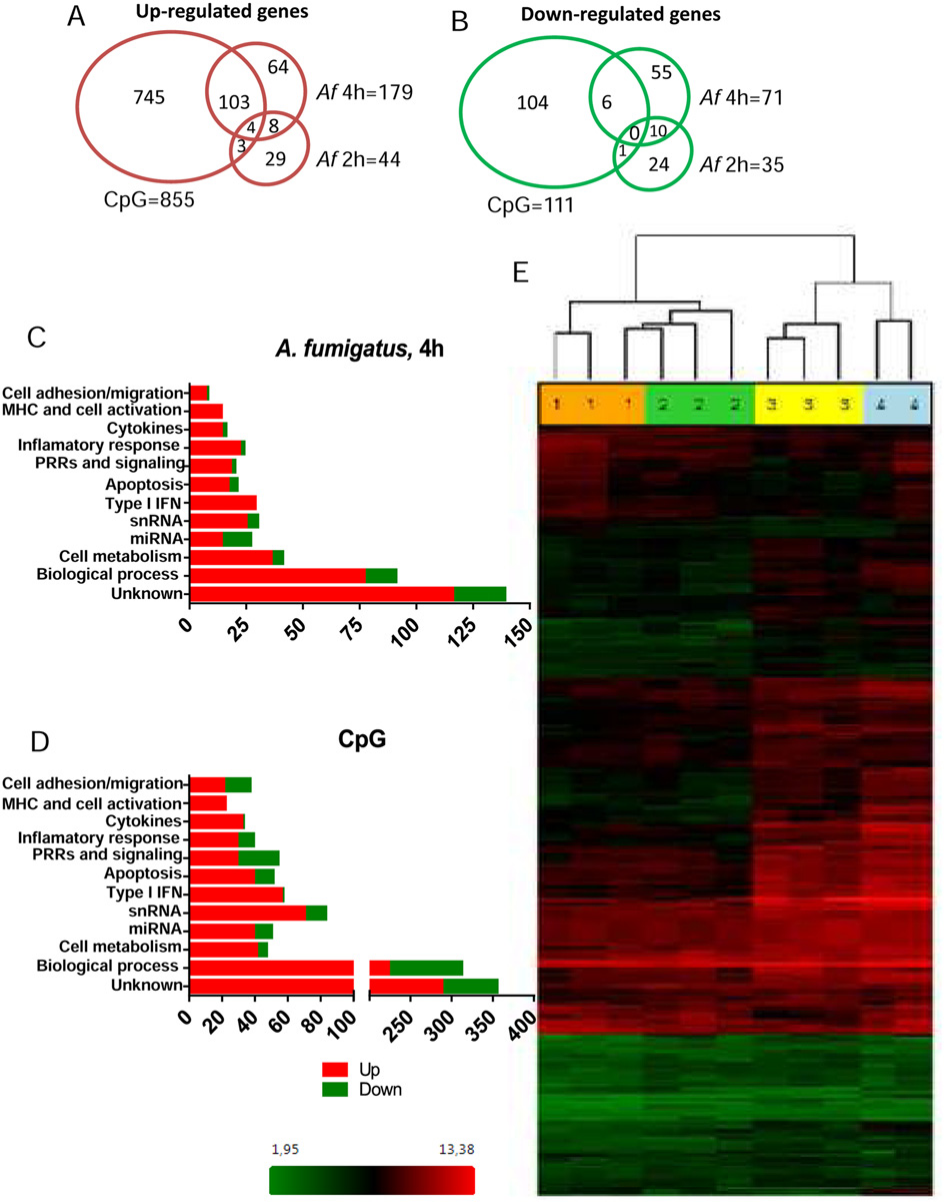
The pDC transcriptome following stimulation with *A*. *fumigatus*. Human pDCs (2 × 10^5^) were left unstimulated or stimulated for 2 or 4 hr with *A. fumigatus* (Af) hyphae (2 × 10^5^). As a positive control, pDCs were stimulated with CpG (20 μg/mL) for 4 hr. The RNA was extracted, converted into cDNA, amplified, labeled, hybridized to microarrays, and analyzed as described in *Methods*. (A, B) Venn diagrams showing the number of up-regulated and down-regulated genes found in each experimental group as well as the overlap between groups. (C, D) Regulated genes classified in categories according to the NetAffx program. (E) A heat map of the 250 genes (see S1 Table) differentially expressed following hyphal or CpG stimulation. Each column represents microarray data from an individual donor’s pDCs that were left unstimulated (number 1, orange columns), Aspergillus-infected for 2 hr (number 2, green columns), Aspergillus-infected for 4 hr (number 3, yellow columns) or CpG-stimulated for 4 hr (number 4, blue columns). The dendrogram above the heat map was generated using Transcriptome Analysis Console Software (TAC) and conveys similarities among pDC samples. Numbers provided with the color spectrum below the heat map are indicative of the linear fold change of each gene. The data are from three donors.

In addition, 966 regulated genes in CpG-stimulated pDCs were found, of which 855 were up-regulated and 111 down-regulated. The Venn diagrams (Fig. 6A and B) show the number of up- and down-regulated genes found in each experimental group as well as the overlap between groups. Regulated genes were classified in immune related categories, cell metabolism and other biological process according to the NetAffx (Affymetrix) program (Fig. 6C and D). A heat map of the 250 genes differentially expressed following 4 hr of hyphal stimulation demonstrates the disparate patterns of gene activation following stimulation with hyphae compared to CpG (Fig. 6E). The hierarchical cluster shows a similar pattern of gene expression among the donors but different patterns of gene expression when comparing the unstimulated, Aspergillus-infected and CpG-stimulated groups. In addition, examination of the heat map reveals a large number of genes which were up-regulated in the 4 hr Aspergillus-infected group and CpG-stimulated group but not in the 2 hr Aspergillus-infected group and unstimulated control group.

Within categories such as innate immune receptors, signaling pathways, cytokine and chemokine production, antigen processing and presentation, and cell activation and migration activation, we next examined which individual genes were up- or down-regulated following a 4 hr hyphal stimulation and compared the fold response to that seen with hyphal stimulation for 2 hr as well as CpG stimulation (Table 1). Two genes encoding C-type lectin receptor expression were up-regulated. The highest expression was found for the CLECL1 gene, which encodes a C-type lectin-like protein (also known as DCAL-1). DCAL-1 is highly expressed by DCs and B cells and may act as a T-cell costimulatory molecule [32]. In addition, the gene CLEC2D, which reportedly encodes a natural killer receptor and is also induced on B cells upon viral infection [33], [34], was also up-regulated. In contrast, the gene CLEC12A, previously reported as a negative regulator of granulocyte and monocyte function that is restricted to immature DCs, was down-regulated in pDCs after Aspergillus infection, suggesting the pDCs were activated [35], [36].

**Table 1:** Dendritic cell activation.

Gene Symbol	Gene description	Fold change		
		Asp 2h	Asp 4h	CpG
***Innate Immune***	***Receptors***			
CLECL1	C-type lectin-like 1	1.78 ± 0.80	10.71 ± 1.28*	***12.44 ± 0.31***
TLR7	Toll-like receptor 7	2.82 ± 0.51	***3.74 ± 0.96***	4.19 ± 1.02
CLEC2D	C-type lectin domain family 2, member D	-1.26 ± 0.43	***2.58 ± 0.07***	***9.81 ± 0.04***
CD180	CD180 molecule	-1.74 ± 0.98	***1.96 ± 0.32***	1.15 ± 0.25
CLEC6A-***Dectin-2***	C-type lectin domain family 6, member A	-1.29 ± 0.28	***-1.89 ± 0.49***	1.66 ± 1.48
CLEC12A	C-type lectin-like 1	***-1.80 ± 0.20***	***-2.49 ± 0.67***	1.24 ± 1.56
CLEC4C	C-type lectin domain family 4, member C	1.30 ± 0.67	***-2.20 ± 0.25***	-2.81 ± 0.62
C5AR1	Complement component 5a receptor 1	-2.70 ± 0.17	***-4.41 ± 0.57***	-2.45 ± 0.81
***Signaling***				
STAT4	Signal transducer and activator of transcription 4	1.67 ± 0.52	***3.07 ± 0.21***	**4.73 ± 0.06**
STAT2	Signal transducer and activator of transcription 2	1.25 ± 0.44	***2.44 ± 0.41***	***2.43 ± 0.02***
IL6ST	Interleukin 6 signal transducer	1.11 ± 0.75	***2.67 ± 0.47***	1.89 ± 0.49
STAT1	Signal transducer and activator of transcription 1	-1.15 ± 0.14	***2.03 ± 0.37***	***1.80*** ± 0.31
MAPKAPK3	mitogen-activated prot kinase-actv prot kinase 3	-1.38 ± 0.36	***-1.98 ± 0.23***	-1.71 ± 0.45
***Cytokines and***	***chemokines***			
CXCL10	Chemokine (C-X-C motif) ligand 10	-1.39 ± 0.97	***5.97 ± 1.04***	***11.01 ± 0.04***
IL2RA	Interleukin 2 receptor, alpha	1.34 ± 0.48	***5.25 ± 0.47***	***14.46 ± 0.13***
TNFSF4	Tumor necrosis fact (ligand) superfam 4	***1.76 ± 0.05***	***5.24 ± 1.33***	***13.55 ± 0.58***
CXCL9	Chemokine (C-X-C motif) ligand 9	-1.81 ± 0.97	***4.71 ± 1.54***	***27.48 ± 0.45***
TNFSF10	Tumor necrosis fact (ligand) superfam 10	-1.00 ± 0.05	***4.42 ± 0.55***	***7.03 ± 0.32***
IL18RAP	Interleukin 18 receptor accessory protein	1.41 ± 0.60	***3.66 ± 1.22***	***20.93 ± 0.17***
CCL22	Chemokine (C-C motif) ligand 22	-1.33 ± 0.23	***3.17 ± 0.48***	1.30 ± 0.21
CCR7	Chemokine (C-C motif) receptor 7	1.19 ± 0.26	***1.81 ± 0.31***	***2.17 ± 0.04***
IL13	Interleukin 13	1.42 ± 0.28	***1,71 ± 0.21***	1.22 ± 0.81
CCL20	Chemokine (C-C motif) ligand 20	-1.41 ± 0.39	***-2.59 ± 0.45***	1.25 ± 0.88
CXCL3	Chemokine (C-X-C motif) ligand 3	-2.44 ± 0.76	***-4.82 ± 0.71***	1.06 ± 0.73
***MHC and cell***	***activation***			
SEC61B	Sec61 beta subunit	***2.47 ± 0.07***	***2.69 ± 0.33***	2.33 ± 0.04
LAMP3	lysosomal-associated membrane protein 3	-1.37 ± 0.42	***2.34 ± 0.72***	***5.74 ± 0.16***
LOC100509457	major histoc complex, class II, DQ alpha 1	1.45 ± 1.15	***2.07 ± 0.33***	1.11 ± 1.10
TAP1	transporter 1, ATP-binding cassette, sub-family B	1.10 ± 0.25	***1.98 ± 0.38***	***2.17 ± 0.11***
TAP2	transporter 2, ATP-binding cassette, sub-family B	-1.1 2± 0.33	***1.72 ± 0.02***	1.52 ± 0.23
***Migration or***	***Adhesion***			
SLC7A11	solute carrier family 7, member 11	1.94 ± 0.82	***6.24 ± 0.55***	***11.41 ± 0.04***
CDH1	cadherin 1, type 1, E-cadherin (epithelial)	1.32 ± 0.51	***4.38 ± 0.33***	1.68 ± 0.43
HAPLN3	hyaluronan and proteoglycan link protein 3	1.05 ± 0.39	***3.42 ± 0.30***	***5.84 ± 0.07***
FSCN1	fascin homolog 1, actin-bundling protein	-1.36 ± 0.55	***3.08 ± 0.30***	***3.92 ± 0.27***
STAP1	signal transducing adaptor family member 1	1.58 ± 0.88	***2.34 ± 0.31***	***3.47 ± 0.34***
SDK2	sidekick cell adhesion molecule 2	-1.27 ± 0.26	***2.64 ± 0.65***	1.30 ± 0.26
VAV2	vav 2 guanine nucleotide exchange factor	-1.20 ± 0.95	***2.53 ± 0.28***	1.91 ± 0.32
SCYL3	SCY1-like 3 (S. cerevisiae)	-1.15 ± 0.43	***2.09 ± 0.41***	***2.37 ± 0.17***
FMNL3	formin-like 3	1.17 ± 0.79	***2.07 ± 0.44***	1.32 ± 0.07
PEAK1	NKF3 kinase family member	-1.11 ± 0.55	***1.86 ± 0.38***	***1.85 ± 0.32***
B4GALT1	UDP-Gal:betaGlcNAc beta1,4-galactosyltransferase	1.05 ± 0.37	***-1.80 ± 0.43***	-1.57 ± 0.03
VAV3	vav 3 guanine nucleotide exchange factor	1.33 ± 0.65	***-2.32 ± 0.66***	-1.86 ± 0.57
VCAN	versican	-2.90 ± 1.18	*-8.50 ± 1.18*	-6.44 ± 1.00

* Significant (*P*<0.05) changes are shown in bold.

Human pDCs were left unstimulated or stimulated with *A. fumigatus* hyphae for 2 hr (Asp 2h), *A. fumigatus* hyphae for 4 hr (Asp 4h), or with CpG for 4 hr (CpG) and then analyzed for gene expression by microarray as in *Methods*. Genes putatively involved in dendritic cell functions (such as innate immune receptors, signaling, cytokine and chemokine production, MHC and cell activation involved genes, and genes involved and migration and adhesion) that had significant changes in the Asp 4 hr group are shown and compared with the other stimulated groups. Data are expressed as fold change compared to unstimulated pDCs and represent the mean ± SD of three donors (except for the CpG group where the data represent the mean of 2 donors).

There were up-regulated genes involved in STAT (Signal Transducers and Activators of Transcription) pathways, including STAT1, STAT2 and STAT4. In response to type I IFN stimulation, STAT1 forms a heterodimer with STAT2 that can bind the ISRE (Interferon-Stimulated Response Element) promoter. Binding the promoter element leads to an increased expression of interferon-stimulated genes (ISGs) [37]. Expression of type I IFN genes markedly increased in response to CpG stimulation but not to Aspergillus infection (Table 2). However, we found several up-regulated genes involved in type I IFN signaling and/or regulation such as IRF2, DHX58, and HERC5. Besides, we found several up-regulated genes known to be induced in response to either IFN-α or IFN-β stimulation (Table 3).

**Table 2:** Genes encoding type I interferons.

Gene Symbol	Gene description	Fold change		
		Asp 2h	Asp 4h	CpG
***Type I Interferons***				
IFNA7	interferon, alpha 7	*1*.*32* ± 0.70	*1*.*32* ± 0.51	*649.33* ± 0.24*
IFNA21	interferon, alpha 21	*1*.*27* ± 0.34	*-1*.*26* ± 0.47	***483.17* ± *0.41***
IFNA4	interferon, alpha 4	*-1*.*23* ± 0.47	*1*.*06* ± 0.46	***427.97* ± *0.08***
IFNA10	interferon, alpha 10	*-1*.*27* ± 0.29	*-1*.*08* ± 0.03	***394.75* ± *0.08***
IFNA17	interferon, alpha 17	*1*.*52* ± 0.81	*1*.*29* ± 0.69	***320.02* ± *0.31***
IFNA1	interferon, alpha 1	*1*.*40* ± 1.63	*-1*.*29* ± 0.92	***287.77* ± *0.15***
IFNA8	interferon, alpha 8	*1*.*13* ± 0.38	*-1*.*16* ± 0.31	***211.06* ± *0.12***
IFNA13	interferon, alpha 13	*-1*.*82* ± 1.16	*-1*.*09* ± 0.37	***202.35* ± *0.03***
IFNA2	interferon, alpha 2	*1*.*27* ± 0.26	*-1*.*26* ± 0.39	***153.96* ± *0.05***
IFNA16	interferon, alpha 16	*1*.*20* ± 0.29	*1*.*30* ± 0.16	***150.52* ± *0.36***
IFNA5	interferon, alpha 5	*1*.*11* ± 0.66	*1*.*28* ± 0.14	***133.71* ± *0.22***
IFNW1	interferon, omega 1	*-1*.*16* ± 0.20	*-1*.*03* ± 0.11	***130.93* ± *0.27***
IFNB1	interferon, beta 1	*-1*.*14* ± 0.14	*-1*.*37*± 0.56	***78.54* ± *0.08***
IFNE	interferon, epsilon	*-1*.*11* ± 0.15	*-1*.*13*± 0.17	***59.90* ± *0.12***
IFNA14	interferon, alpha 14	*-1*.*47* ± 0.64	*-1*.*29*± 0.48	***32.99* ± *0.01***

* Significant (*P*<0.05) changes are shown in bold.

As in Table 1 except genes encoding type I IFNs are shown.

**Table 3:** Type I Interferon-induced genes.

Gene Symbol	Gene description	Fold change		
		Asp 2h	Asp 4h	CpG
***Type I IFN induced genes***				
IFI44L *	interferon-induced protein 44-like	1.34 ± 0.26	***6.09 ± 0.58^#^***	***8.90 ± 0.47***
USP18	ubiquitin specific peptidase 18	-1.09 ± 0.20	***5.29 ± 0.26***	***8.64 ± 0.06***
SAMD9L	sterile alpha motif domain containing 9-like	-1.32 ± 0.47	***4.22 ± 0.35***	***9.31 ± 0.12***
OAS2 *	2′-5′-oligoadenylate synthetase 2, 69/71kDa	-1.15 ± 0.12	***3.97 ± 0.38***	***4.82 ± 0.03***
XAF1	XIAP associated factor 1	-1.31 ± 0.17	***3.46 ± 0.65***	***5.16 ± 0.07***
EIF2AK2 *	eukaryotic translation initiation factor 2-alpha kinase	1.05 ± 1.06	***3.29 ± 0.69***	***3.58 ± 0.10***
EBI3	Epstein-Barr virus induced 3	-1.02 ± 0.67	***4.77 ±1.21***	***8.88 ± 1.25***
HERC5*	HECT and RLD domain containing E3 ubiquitin protein ligase 5	1.17 ± 0.72	***3.28 ± 0.37***	***9.40 ± 0.14***
STAMBPL1*	STAM binding protein-like 1	1.21 ± 0.62	***3.21 ± 0.98***	***2.27 ± 0.78***
IFIT1	interferon-induced protein with tetratricopeptide repeats 1	-2.07 ± 0.16	***3.00 ± 0.59***	***8.04 ± 0.07***
IFI16*	interferon, gamma-inducible protein 16	1.55 ± 0.35	***2.94 ± 0.34***	***3.91 ± 0.28***
OAS3 *	2′-5′-oligoadenylate synthetase 3, 100kDa	-2.06 ± 0.47	***2.74 ± 0.49***	***3.92 ± 0.13***
OAS1 *	2′-5′-oligoadenylate synthetase 1, 40/46kDa	1.06 ± 0.10	***2.69 ± 0.33***	***3.52 ± 0.05***
IFI6	interferon, alpha-inducible protein 6	1.02 ± 0.52	***2.68 ± 0.26***	***8.53 ± 0.45***
PML *	promyelocytic leukemia	-1.24 ± 0.51	***2.65 ± 0.12***	***4.30 ± 0.36***
RSAD2*	radical S-adenosyl methionine domain cont. 2	-1.43 ± 0.06	***2.60 ± 0.74***	***9.26 ± 0.31***
DDX60*	DEAD (Asp-Glu-Ala-Asp) box polypeptide 60	-1.63 ± 0.81	***2.59 ± 0.41***	***7.40 ± 0.24***
IRF2	interferon regulatory factor 2	-1.12 ± 0.50	***2.44 ± 0.47***	***4.38 ± 0.69***
IFI44 *	interferon-induced protein 44	-1.70 ± 0.45	***2.36 ± 0.65***	***2.85 ± 0.11***
SP100 *	SP100 nuclear antigen	-1.15 ± 0.47	***2.16 ± 0.16***	***2.52 ± 0.12***
ADAR *	adenosine deaminase, RNA-specific	1.16 ± 0.19	***1.94 ± 0.45***	***1.95 ± 0.25***
DHX58	DEXH (Asp-Glu-X-His) box polypeptide 58	-1.23 ± 0.30	***1.86 ± 0.20***	***5.12 ± 0.33***
IFI35 *	interferon-induced protein 35	-1.11 ± 0.51	***1.86 ± 0.45***	***2.74 ± 0.63***
MX1*	Myxovirus resistance 1, ifn-inducible protein p78	1.10 ± 0.31	***1.83 ± 0.26***	***2.06 ± 0.27***
IFIT5	ifn-induced protein tetratricopeptide repeats 5	-1.29 ± 0.21	***1.72 ± 0.29***	***4.50 ± 0.18***

*Genes previously described as regulated in viral infection.

^*#*^ Significant (*P*<0.05) changes are shown in bold.

As in Table 1 except type I IFN-induced genes are shown.

In addition, the gene MAPKAP3, involved in the TLR signaling pathway, was down-regulated, although TLR7 gene expression was up-regulated upon Aspergillus stimuli. The expression of pDC genes involved in cytokine and chemokine production changed following hyphal stimulation. While the expression of CXCL10, CXCL9, CCR7 and CCL22 was up-regulated, the expression of CXCL3 and CCL20 was down-regulated. In addition, two TNF cytokine family genes were at higher levels in the Aspergillus-infected samples compared with the unstimulated pDCs.

Following antigen recognition and phagocytosis, DCs process antigen and usually migrate to the lymph nodes where the antigen is presented to naive T cells. After Aspergillus infection, the pDCs up-regulated some genes involved in antigen processing and presentation via MHC such as LAMP3, TAP1, TAP2 and SEC16B. Moreover, several genes involved in cell shape, spreading control, cell adhesion and migration were regulated as well (Table 1). Finally, the transcriptome profile of Aspergillus-infected pDCs included many regulated genes involved in apoptosis (Table 4).

**Table 4:** Apoptosis and regulation.

Gene Symbol	Gene description	Fold change		
		Asp 2h	Asp 4h	CpG
***Apoptosis***				
CD38	CD38 molecule	1.07 ± 0.55	3.96 ± 0.27*	***6.01 ± 0.01***
BCL2L1	BCL2-like 1	-1.22 ± 0.56	***2.54 ± 0.61***	***3.95 ± 0.41***
ARHGEF17	Rho guanine nucleotide exchange factor (GEF) 17	1.06 ± 0.04	***1.96 ± 0.69***	1.08 ± 0.05
MLLT11	myeloid/lymphoid or mixed-lineage leukemia	1.54 ± 0.40	***1.94 ± 0.19***	***2.29 ± 0.04***
FAS	Fas (TNF receptor superfamily, member 6)	-1.12 ± 0.25	***1.85 ± 0.42***	1.46 ± 0.53
ARHGEF3	Rho guanine nucleotide exchange factor (GEF) 3	1.40 ± 0.53	***1.75 ± 0.14***	***1.97 ± 0.32***
ANXA1	annexin A1	-1.20 ± 0.03	***-1.81 ± 0.32***	1.12 ± 0.25
CEBPB	CCAAT/enhancer binding protein (C/EBP), beta	-1.47 ± 0.26	***-2.02 ± 0.33***	-1.17 ± 0.42
SOX4	SRY (sex determining region Y)-box 4	1.92 ± 1.13	***-2.10 ± 1.17***	-1.97 ± 0.13
JMY	junction mediating and regulatory protein, p53	-1.21 ± 0.46	***-2.53 ± 0.31***	-2.10 ± 0.06
TNFSF14	TNF (ligand) superfamily, member 14	-2.07 ± 0.50	***-3.42 ± 0.29***	-1.24 ± 0.81
***Regulation***				
CALCRL	calcitonin receptor-like	1.15 ± 1.09	***7.37 ± 0.79***	***13.53 ± 0.70***
TRAFD1	TRAF-type zinc finger domain containing 1	1.48 ± 0.14	***5.23 ± 0.57***	***6.88 ± 0.06***
FCRL3	Fc receptor-like 3	-1.12 ± 1.03	***2.18 ± 0.44***	1.75 ± 0.43
ENDOG	endonuclease G	1.12 ± 0.36	***1.80 ± 0.30***	1.49 ± 0.17
CD200	CD200 molecule	-1.67 ± 0.76	***1.83 ± 0.40***	3.20 ± 0.19

* Significant (*P*<0.05) changes are shown in bold.

As in Table 1, except genes associated with apoptosis and regulation are shown.

## Discussion

Several PRRs have been reported to recognize ligands on *A*. *fumigatus* including Dectin-1 (β-glucan), DC-SIGN (galactomannans), and Dectin-2 (α-mannan) [38], [39]. TLR2 and TLR4 also participate in signaling responses against this fungus [40]. The recognition receptors expressed on pDCs have not been well studied. Human pDCs have been shown to express Dectin-2, Siglec-H and DC immunoreceptor (DCIR), but not Dectin-1, mannose receptor, DC-SIGN, Mincle, TLR2 and TLR4 [24], [25], [41], [42]. In addition, human pDCs express some complement and Fc receptors [43]. In the present study, we demonstrated that Dectin-2 is involved in the recognition of *A*. *fumigatus* hyphae by human pDCs and that this recognition leads to TNF-α and IFN-α release as well as enhanced antifungal activity by pDCs. While human pDCs express TLR9, we previously demonstrated that release of these cytokines following hyphal stimulation occurred in a TLR9-independent manner [19]. Although Dectin-1 is involved in the recognition of *A*. *fumigatus* ligands by other cell types [22], [23] we did not find evidence of its involvement in pDC. The putative involvement of murine Dectin-2 in the release of IFN-α was showed by Seeds et al. [44]. Mannan, a broad blocking reagent against mannose receptors including Dectin-2, inhibited murine pDC IFN-α production in response to inactivated influenza virus. Similar to our findings with human pDCs, an anti-Dectin-1 monoclonal antibody had no effect on IFN-α production by pDC. Moreover, experiments with transfected B3Z cells indicate that Dectin-2 works in cooperation with FcRγ to trigger signaling responses against *A*. *fumigatus* hyphae. Similar cooperative interactions between Dectin-2 and FcRγ have been demonstrated using zymosan [26]. In addition, it was recently demonstrated that hyphae stimulated increased IL-17RC expression in neutrophils in a Dectin-2-dependent manner [45]. Taken together, our data strongly support a central role for pDC Dectin-2 in hyphal recognition, antifungal activity and cytokine release.

Notably, when the pDCs were treated either with mannan or anti-Dectin-2 antibody, their association with hyphae was only decreased by about half. This suggests that Dectin-2 is not the only PRR participating in the recognition of *A*. *fumigatus* hyphae by human pDCs. Future studies will be needed to determine the identity of the other receptors involved. In addition to Dectin-2, numerous candidate receptors are expressed by pDCs, including Siglec-H and DC immunoreceptor (DCIR) [20], [25], [41]. Interestingly, in the microarray experiments, we found that following *A*. *fumigatus* stimulation of pDCs, the highest up-regulated gene encodes for CLECL1 (also known as DCAL-1). Similarly to Dectin-1 and Dectin-2, CLECL1 is a C-type lectin molecule. CLECL1 expression reportedly is restricted to hematopoietic cells, including pDCs [32]. However, it is unknown whether CLECL1 functions as a recognition receptor. A caveat to interpretation of the microarray studies is gene expression of the receptors responsible for hyphae recognition may not be up-regulated following contact of pDCs with *A*. *fumigatus*. Indeed, we found this was the case for Dectin-2 as up-regulation of Dectin-2 expression was not seen following fungal stimulation.

In the absence of activating signals, pDCs reportedly undergo spontaneous apoptosis [46], [47]. Our previous report demonstrated that the interaction between human pDCs and *A*. *fumigatus* hyphae results in the accelerated death of the pDCs by a mechanism that was partly mediated by fungal gliotoxin secretion but still resulted in antifungal activity [19]. Thus, we asked if the recently described mechanism of cell death known as ETosis, largely described in neutrophils [48] but also reported in other cell types, occurred following the recognition of *A*. *fumigatus* hyphae by human pDCs. The dying cells form ETs composed of chromatin decorated with antimicrobial proteins that are able to trap and kill pathogens, including bacteria and fungi, and thus, contribute to extracellular anti-microbial host defense [49], [50], [51]. The different ETs have several features in common, regardless of the type of cells from which they originated, including a DNA backbone with embedded antimicrobial peptides, proteases, and citrullinated histones [7]. The morphotype of the pathogen also appears to influence NET formation. A recent study found that while large hyphae of *C*. *albicans* induced NETosis, a mutant of *C*. *albicans* that is unable to form hyphae failed to induce NETosis [27]. In our study, following incubation of pDCs with *Aspergillus* hyphae, many pDCs that spread over hyphae had disrupted DNA and stained strongly positive for citrullinated histone H3. On the other hand, unstimulated pDCs had intact nuclear DNA with no detectable staining with antibodies directed at citrullinated histone H3. These observations suggest that pET formation occurs by mechanisms similar to that described for other types of immune cells, including chromatin decondensation mediated by histone citrullination [52]. Histone hypercitrullination mediates chromatin decondensation and NET formation. When the interactions of pDCs with hyphae were examined by SEM, areas colonized by *A. fumigatus* showed ET structures that engulfed fungal surfaces. The appearance of pETs is very similar to that described for NETs [29], [30], [31]. A recent study reported that <5% of neutrophils undergo NETosis following incubation with Candida albicans hyphae [27], which is somewhat comparable to our observation that ~1% of pDCs form pETs following incubation with Aspergillus hyphae. While these studies establish that ET formation occurs following stimulation of pDCs with *A*. *fumigatus* hyphae, future studies will be needed to determine whether pETs contribute to the antifungal activity of pDCs. Antifungal effects of NETs, albeit not robust, have been reported for *A*. *fumigatus* [8], [30], [31].

To better understand the full role of pDC in antifungal defenses, we took an unbiased systems biology approach and investigated the pDC transcriptome profile upon *A*. *fumigatus* infection. Comparing 2 and 4 hr time points after infection, we found more genes were regulated at the latter time point. Unfortunately, limitations on yields of pDCs from individual blood donors precluded examination of additional time points. Of interest, several genes that were initially described as being involved in viral infections or virus-induced leukemia [53], [54], [55], [56], [57] were up-regulated in pDCs both after CpG stimulation and *A*. *fumigatus* infection. The expression of several genes involved in dendritic cell activation, chemokine production, and antigen presentation and processing supports the hypothesized involvement of pDC in host defense against *A*. *fumigatus*. In addition, many genes involved in the apoptotic process were also up-regulated after both CpG stimuli and *A*. *fumigatus* infection. Our previous article presented two lines of evidence strongly suggesting that the high rate of pDC cytotoxicity following incubation with *A. fumigatus* hyphae is at least partially due to secreted factors released by the fungi. First, pDC cytotoxicity was observed when the pDCs and hyphae were separated by a transwell. Second, pDC cytotoxicity was significantly reduced following incubation with hyphae from *A. fumigatus* strains genetically engineered to be deficient in gliotoxin production [19]. Finally, while early apoptotic cells normally preserve their cell membrane integrity, apoptosis can also progress to secondary necrosis and membrane leakage [58]. Thus, apoptotic gene up-regulation presumably is contributing to the antifungal activity of the dying pDCs and to pET formation as presented in this report.

We previously showed that small quantities of IFN-α are released by pDCs upon stimulation with *A*. *fumigatus* hyphae and that mice null for the type I IFN receptor are hypersusceptible to intravenous *A*. *fumigatus* challenge [19]. In the present study, we confirmed that IFN-α is released by pDCs upon stimulation with *A*. *fumigatus* hyphae. While we did not see significant up-regulation of genes encoding type I IFNs by *A*. *fumigatus-*infected pDCs, we did see up-regulation of numerous type I IFN-induced genes. This suggests that hyphae-induced type I IFN release is regulated post-transcriptionally [59], [60] or the microarrays lack the sensitivity to detect relatively small changes in gene expression. In contrast, CpG robustly stimulated up-regulation of genes encoding type I IFNs.

Therefore, our data show for the first time that: 1) Dectin-2 participates in the recognition of *A*. *fumigatus* hyphae by pDCs, 2) the interaction between pDCs and *A*. *fumigatus* results in the formation of pETs, and 3) a distinctive transcriptional profile is seen following stimulation of pDCs by hyphae. These data add significantly to our knowledge of how pDCs contribute to host defenses in non-viral infections. The challenge will be to apply these findings to infected patients.

## Materials and Methods

### Ethics statement

All research involving human participants was approved by the University of Massachusetts Medical School’s Institutional Review Board. Written informed consent was obtained from all human participants and all clinical investigations were conducted according to the principles expressed in the Declaration of Helsinki.

### Reagents and cell culture

RPMI-1640 was obtained from GIBCO (Invitrogen). pDC and Aspergillus media consisted of RPMI-1640 supplemented with 100 U/ml penicillin, 100 U/ml streptomycin, 2 mM L-glutamine, 0.5 mM HEPES, and 1 mM sodium pyruvate. Mannan, laminarin, dextran (molecular weight 473,000) and zymosan were purchased from Sigma-Aldrich. Rat monoclonal anti-Dectin-1 and anti-Dectin-2 blocking antibodies were obtained from Serotec and R&D Systems, respectively. The immunostimulatory CpG 2336 oligonucleotide was synthesized with phosphothioate linkages by Integrated DNA Technologies.

### *A*. *fumigatus* strains and culture

The wild-type *A. fumigatus* clinical isolate Af293 [61] was obtained from the Fungal Genetics Stock Center. Cultivation of *A. fumigatus*, harvesting of conidia and growth into swollen conidia and hyphae were performed as in our previous studies with slight modifications [40], [19]. Briefly, fungi were grown on Sabouraud Dextrose Agar slants and conidia were harvested with PBS containing 0.05% Tween 20. The conidia were then vortexed, filtered through a 30-μm nylon mesh, washed, counted and stored in water at 4°C for up to a week. To generate hyphae, conidia were incubated at 21°C for 16 hr in Aspergillus media to swell the conidia, and then an additional 3 hr at 37°C to promote germination.

### Isolation of human pDCs

Human pDCs were isolated from healthy donors as described [19], [62]. Peripheral blood was collected by venipuncture. The blood was anticoagulated with heparin, and the peripheral blood mononuclear cells (PBMCs) were purified by Ficoll-Hypaque density gradient centrifugation. Highly purified human pDCs were obtained from PBMC by two rounds of positive selection using CD304-coated magnetic beads (Miltenyi Biotec, cat 130–090–532) [19]. For the XTT and cytokine release studies, highly purified human pDCs were obtained from PBMC by negative selection using magnetic beads (Miltenyi Biotec, cat 130–097–145). No contaminating PMNs were observed.

### pDC and *A. fumigatus* hyphae association

*A. fumigatus* conidia (5 × 10^4^) were plated in flat-bottom 96-well half area plates and grown in Aspergillus media to hyphae of 10–20 μm average length. pDCs (5 × 10^4^) were then added to the hyphae in a final volume of 100 μl pDC media for 2 hr at 37°C. The pDCs were previously treated with laminarin (0.5 mg/mL), yeast mannan (1 mg/mL) or both for 30 min. Dextran (0.5 mg/mL) was used as an irrelevant polysaccharide control. Additional experiments were performed by using anti-Dectin-1 (0.1 mg/mL) and anti-Dectin-2 (0.1 mg/mL) blocking antibodies. Binding was quantified using an inverted microscope (Zeiss) by counting number of human pDCs tightly associated with hyphae in 10 different fields. The cell association index was then calculated by dividing the number of pDCs in association with hyphae by the total number of pDC counted and multiplying this fraction by 100.

### XTT assay of antifungal activity

Antifungal activity was measured by the XTT assay as described [19], [63]. Briefly, *A. fumigatus* conidia (5 × 10^3^) were plated in 96-well, half-area plates and grown in pDC media to hyphae of 10–20 μm average length. pDCs (5 × 10^4^) were left untreated or incubated with anti-Dectin-2 antibody for 30 minutes at 4°C and then added to the hyphae in a final volume of 100 μl pDC media. Control wells contained hyphae but no pDCs. Following 2 hr incubation, the pDCs were subjected to hypotonic lysis by three gentle washes with distilled water followed by a 30 min incubation with distilled water at 37°C. Supernatants then were removed, with great care taken not to remove the hyphae. pDC media containing 400 μg/ml of XTT and 50 μg/ml of Coenzyme Q, were added, and the wells were incubated for 2 hr at 37°C. The OD_450_ and OD_650_ were then measured, and data were expressed as the percent of antifungal activity according to the published formula [19].

### Cytokine release

*A. fumigatus* conidia (5 × 10^4^) were plated in 96-well plates and grown in pDC media to hyphae of 10–20 μm average length. pDCs (5 × 10^4^) were left untreated or incubated with anti-Dectin-2 or anti-Dectin-1 antibodies for 30 minutes at 4°C. pDCs were then added to the hyphae in a final volume of 200 μl pDC media containing voriconazole (0.5 μg/mL) to inhibit fungal overgrowth. Control wells contained pDCs only, pDCs and antibodies, or pDC and CpG. After 6 hr of incubation at 37°C, the supernatants were removed and TNF-α and IFN-α levels were measured by ELISA according to the manufacturers’ protocols (eBioscience for TNF-α; PBL Assay Science for IFN-α).

### B3Z cells and β-galactosidase activity

Transgenic cell lines were used to assess the involvement of Dectin-2 and FcRγ in the recognition of *A*. *fumigatus* hyphae. The hybridoma T cell line, B3Z has a reporter for nuclear factor of activated T-cells (NFAT) driven activation of β-galactosidase [64]. B3Z cells were retrovirally transduced with murine Dectin-2, wild-type FcRγ chain, Dectin-2 and wild-type FcRγ chain, or Dectin-2 and a signaling-deficient mutant of FcRγ chain as described by Robinson et al., 2009 [26]. These cell lines were a gift from Caetano Reis e Souza (Immunobiology Laboratory, Cancer Research UK, London Research Institute, England, UK) and obtained from Marcel Wϋethrich (University of Wisconsin, Madison). *A. fumigatus* hyphae (1 × 10^5^) or conidia (1 × 10^5^) were incubated in 48 well plates with each of the BZ3-derived cells (2 × 10^5^) in RPMI media for 2 hr at 37°C. Control wells contained BZ3-derived cells only and were left unstimulated or were stimulated with zymosan (100 μg/mL). NF-AT activation was measured using a β-galactosidase assay. Media were removed from each well and replaced with 100 μl buffer (PBS, 0.05% Triton X-100, 2 mM magnesium sulfate) followed by incubation for 30 min at 4°C. 50 μl of each lysate were transferred to a well of a 96 well black plate and mixed with 1 ul of 10 mM 4-Methylumbelliferyl β-D-galactoside. Relative fluorescence intensities (RFU’s) were measured using a fluorescence microplate reader (Tecan GENios) at 5 min intervals for 1 hr at 37°C. β-galactosidase activity was calculated at its maximum rate as RFU/min.

### Confocal microscopy

Circular tissue culture slides (13 mm diameter) were pretreated with 1% Poly-L-lysine solution (Sigma-Aldrich) and placed in 24-well plates. *A. fumigatus* conidia (2 × 10^5^) were then added to the wells and germinated in Aspergillus media to hyphae of 10–20 μm average length. The wells were gently washed with PBS and the pDCs (2 × 10^5^) were then added to the hyphae in a final volume of 500 μl pDC media and incubated for 4 or 6 hr at 37°C. The samples were fixed with 2% buffered paraformaldehyde and washed three times with PBS. For immunostaining, specimens were treated as described previously [49]. Briefly, specimens were washed 3 times with PBS, permeabilized for 10 min using 0.5% Triton X-100 in PBS and washed again 3 times with PBS. Subsequently, the samples were blocked with 3% cold water fish gelatin, 5% donkey serum, 1% BSA (w/V), 0.25% Tween 20 in PBS (blocking solution) for 30 min at room temperature, and incubated with primary antibodies directed against histone H3 (citrulline R2 + R8 + R17; ab5103, Abcam) and human CD123 (clone 6H6, eBioscience) diluted in blocking solution overnight at 4°C. After 3 washing steps with PBS, primary antibodies were detected with species-specific secondary antibodies coupled to Alexa Fluor 488- and 568-conjugated secondary antibodies (Life Technologies) diluted in blocking solution, respectively. DNA was visualized with 4′,6-diamidino-2-phenylindole (DAPI; Life Technologies) and slides were mounted with fluorescence mounting medium (Dako). Images were captured with a C1 plus confocal microscope (Nikon Instruments) and a 60x oil immersion objective using the operating software EZ-C1 3.91 (Nikon Instruments). Wavelengths of 405 nm (diode), 488 nm (Argon), and 543 nm (HeNe) were used to excite DAPI, Alexa Fluor 488 (and transmission images), and Alexa Fluor 568, respectively. Images were captured in separate passes to avoid cross talk and are presented as maximum intensity projections from Z-stacks. All images were slightly adjusted for background fluorescence and signal intensity in NIS elements software AR 3.2 (Nikon Instruments).

### High resolution SEM analysis of pET fine structure

*A*. *fumigatus* conidia (4 × 10^5^ *)* were plated in 18 mm cover slips in 12-well plates and grown in *Aspergillus* media to hyphae of 10–20 μm average length. pDCs (2 × 10^5^) were then added to the hyphae in a final volume of 1 mL pDC media for 2 and 4 hr at 37°C. After fixation with 2.5% (v/v) glutaraldehyde in 0.1 M sodium cacodylate buffer, pH 7.2 for 1 hr at room temperature, specimens were contrasted using repeated changes of 0.5% OsO_4_ in dH2O and 0.05% tannic acid. Specimens were then rinsed in dH2O and dehydrated through a graded series to 100% ethanol and then critical point dried in liquid CO_2_. The cover slips with the specimens were affixed with carbon tape to the surface of SEM aluminum stubs and first coated with 30 nm of carbon, and further sputter coated with Au/Pd (80/20). The specimens were examined using a FEI Quanta 200FEG MK II scanning electron microscope at 10Kv accelerating voltage. Areas containing pET-like structures were recorded at high magnification.

### RNA extraction and microarray experiments

*A. fumigatus* conidia (2 × 10^5^) were plated in 48-well plates and grown in pDC media to hyphae of 10–20 μm average length. pDCs (2 × 10^5^) were then added to the hyphae in a final volume of 300 μl pDC media for 2 and 4 hr at 37°C. Unstimulated and CpG-stimulated pDCs were incubated for 2 hr and 4 hr, respectively. The total RNA was extracted with the RNeasy Mini Kit (Qiagen, Hilden, Germany). The quantity of total RNA was measured with a spectrophotometer at 260 nanometers, and the RNA integrity was assessed using an RNA 6000 Nano LabChip Kit on an Agilent 2100 Bioanalyzer (Agilent Technologies, Palo Alto, CA, U.S.A.). Total RNA (80–500 ng) was reverse transcribed and the single stranded cDNA was amplified using the Ambion WT Expression Kit (Life Technologies, Inc.). The purified cDNA (5.5 μg) was subsequently fragmented and labeled using the GeneChip WT Terminal Labeling Kit (Affymetrix Inc., Santa Clara, CA, U.S.A.). Labeled cDNA (3.5 ug) was then hybridized to the GeneChip Human Gene 2.0 ST array (Affymetrix, Inc.) using the GeneChip Hybridization Oven 640 (Affymetrix, Inc.) at 60 rotations per minute at 45°C for 16–18 hrs. After hybridization, the arrays were washed and stained according to the Affymetrix protocol using a GeneChip Fluidics Station 450 (Affymetrix). The arrays were scanned using the GeneChip Scanner 3000 (Affymetrix). The data were analyzed using Express Console and Transcriptome Analysis Console (TAC) software (Affymetrix, Inc.). The regulated genes were calculated by dividing the linear intensity value found for each probe from experimental groups (2 or 4 hr of pDC-*A*. *fumigatus* interaction and CpG-stimulated pDCs) by the linear intensity value found for each probe from the control group (unstimulated pDCs). We considered 1.7 linear fold changes the cut-off to classify the down-regulated and up-regulated genes [65]. Microarray data were deposited in NCBI under GEO accession number GSE55467.

### Statistical analysis

For comparisons of two groups, means ± standard errors were analyzed by the two-tailed unpaired Student *t*-test with the Bonferroni correction applied when making multiple comparisons. For comparisons of greater than two groups, significance was determined using the one- or two-way analysis of variance (ANOVA) with Tukey’s multiple correction. Calculations were performed using statistical software (GraphPad Prism 5). Statistical significance was defined as *P*<0.05 following corrections. For the microarray analysis, Transcriptome Analysis Console (TAC) software (Affymetrix, Inc.) was used.

## Supporting Information

S1 Table Genes significantly up- or down-regulated in pDCs after 4 hr of stimulation with *A. fumigatus* hyphae. Human pDCs were left unstimulated or stimulated with *A. fumigatus* hyphae for 2 hr (Asp 2h), *A. fumigatus* hyphae for 4 hr (Asp 4h), or with CpG for 4 hr and then analyzed for gene expression by microarray as in *Methods*. The complete set of 250 genes that had significant changes in the Asp 4h group is shown along with the other stimulated groups. Data are expressed as fold change compared to unstimulated pDCs and represent the mean among three donors, (except for CpG group where the data represent the mean between two donors). Significant (*P*<0.05) changes are shown in bold. Note that some genes are also included in Tables 1–4.
(DOCX)
