# Supplementary material for: Recognition of Aspergillus fumigatus Hyphae by Human Plasmacytoid Dendritic Cells Is Mediated by Dectin-2 and Results in Formation of Extracellular Traps
Source: PLoS Pathog. 2015 Feb 6;11(2):e1004643. doi: 10.1371/journal.ppat.1004643 (PMC4450068; doi:10.1371/journal.ppat.1004643)
Supplement: S1 Table — Human pDCs were left unstimulated or stimulated with A. fumigatus hyphae for 2 hr (Asp 2h), A. fumigatus hyphae for 4 hr (Asp 4h), or with CpG for 4 hr and then analyzed for gene expression by microarray as in Methods. The complete set of 250 genes that had significant changes in the Asp 4h group is shown along with the other stimulated groups. Data are expressed as fold change compared to unstimulated pDCs and represent the mean among three donors, (except for CpG group where the data represent the mean between two donors). Significant (P<0.05) changes are shown in bold. Note that some genes are also included in Tables 1–4. (DOCX) [file ppat.1004643.s001.docx]

| **Table S1 - Genes significantly up- or down-regulated in pDCs after 4h of stimulation with *A. fumigatus* hyphae.** | | | | |
| --- | --- | --- | --- | --- |
| **Gene Symbol** | **Gene description** | ***Asp 2h*** | ***Asp 4h*** | ***CpG*** |
| CLECL1 | C-type lectin-like 1 | 1.78 | ***10.71**** | ***12.44*** |
| CALCRL | calcitonin receptor-like | 1.15 | ***7.37*** | ***13.53*** |
| SLC7A11 | solute carrier family 7, member 11 | 1.94 | ***6.24*** | ***11.41*** |
| IFI44L | interferon-induced protein 44-like | 1.34 | ***6.09*** | ***8.90*** |
| CXCL10 | Chemokine (C-X-C motif) ligand 10 | -1.39 | ***5.97*** | ***11.01*** |
| USP18 | ubiquitin specific peptidase 18 | -1.09 | ***5.29*** | ***8.64*** |
| IL2RA | Interleukin 2 receptor, alpha | 1.34 | ***5.25*** | ***14.46*** |
| TRAFD1 | TRAF-type zinc finger domain containing 1 | 1.48 | ***5.23*** | ***6.88*** |
| TNFSF4 | Tumor necrosis fact (ligand) superfam, member 4 | ***1.76*** | ***5.24*** | ***13.55*** |
| 16722401 | Unknown or uncharacterized | 2.75 | ***5.16*** | 1.68 |
| CXCL9 | Chemokine (C-X-C motif) ligand 9 | -1.81 | ***4.71*** | ***27.48*** |
| CANX | calnexin (CANX), transcript variant 2, mRNA. | 1.26 | ***4.80*** | 1.56 |
| TNFSF10 | Tumor necrosis fact (ligand) superfam, member 10 | -1.00 | ***4.42*** | ***7.03*** |
| CDH1 | cadherin 1, type 1, E-cadherin (epithelial) | 1.32 | ***4.38*** | 1.68 |
| HESX1 | HESX homeobox 1 | 1.23 | ***4.37*** | ***10.82*** |
| GPR180 | G protein-coupled receptor 180 | 1.71 | ***4.27*** | ***5.41*** |
| 16653559 | Unknown or uncharacterized | 1.19 | ***4.25*** | 1.63 |
| SAMD9L | sterile alpha motif domain containing 9-like | -1.32 | ***4.22*** | ***9.31*** |
| 17123266 | Unknown or uncharacterized | -1.53 | ***4.04*** | ***7.68*** |
| OAS2 | 2'-5'-oligoadenylate synthetase 2, 69/71kDa | -1.15 | ***4.00*** | ***4.82*** |
| CD38 | CD38 molecule | 1.07 | ***3.96*** | ***6.01*** |
| RGL1 | ral guanine nucleotide dissociation stimulator-like 1 | 1.07 | ***3.95*** | ***5.43*** |
| IGFBP4 | insulin-like growth factor binding protein 4 | 1.18 | ***3.85*** | ***13.68*** |
| TLR7 | Toll-like receptor 7 | 2.82 | ***3.74*** | 4.19 |
| TSPAN33 | tetraspanin 33 | -1.24 | ***3.66*** | ***2.25*** |
| IL18RAP | Interleukin 18 receptor accessory protein | 1.41 | ***3.66*** | ***20.93*** |
| IKZF2 | IKAROS family zinc finger 2 (Helios) | 1.19 | ***3.64*** | ***10.17*** |
| TBC1D13 | TBC1 domain family, member 13 | 1.07 | ***3.55*** | ***2.54*** |
| PAG1 | phosphoprotein associated with glycosphingolipid | 1.28 | ***3.53*** | 1.91 |
| XAF1 | XIAP associated factor 1 | -1.31 | ***3.46*** | ***5.16*** |
| HAPLN3 | hyaluronan and proteoglycan link protein 3 | 1.05 | ***3.42*** | ***5.84*** |
| HAPLN3 | hyaluronan and proteoglycan link protein 3 | 1.05 | ***3.42*** | ***5.84*** |
| 17123264 | Unknown or uncharacterized | -1.34 | ***3.40*** | ***6.43*** |
| SLC38A5 | solute carrier family 38, member 5 | 1.50 | ***3.31*** | ***3.14*** |
| EIF2AK2 | eukaryotic translation initiation factor 2-alpha kinase 2 | 1.05 | ***3.29*** | ***3.58*** |
| EBI3 | Epstein-Barr virus induced 3 | -1.02 | ***3.28*** | ***8.88*** |
| HERC5 | HECT and RLD domain containing E3 ubiquitin protein ligase 5 | 1.17 | ***3.28*** | ***9.40*** |
| C22orf28 | chromosome 22 open reading frame 28 | 1.35 | ***3.27*** | ***3.57*** |
| TARP | TCR gamma alternate reading frame protein | 5.05 | ***3.25*** | ***5.95*** |
| TRANK1 | tetratricopeptide repeat and ankyrin repeat containing 1 | -1.35 | ***3.23*** | ***5.28*** |
| 17123262 | Unknown or uncharacterized | -1.92 | ***3.21*** | ***5.51*** |
| STAMBPL1 | STAM binding protein-like 1 | 1.21 | ***3.21*** | 2.27 |
| CCL22 | Chemokine (C-C motif) ligand 22 | -1.33 | ***3.17*** | 1.30 |
| 16664332 | Unknown or uncharacterized | -1.02 | ***3.14*** | 1.17 |
| NBEAL1 | 60S ribosomal protein L12-like neurobeachin-like 1 | 1.14 | ***3.08*** | ***1.95*** |
| FSCN1 | fascin homolog 1, actin-bundling protein | -1.36 | ***3.08*** | ***3.92*** |
| STAT4 | Signal transducer and activator of transcription 4 | 1.67 | ***3.07*** | ***4.73*** |
| IFIT1 | interferon-induced protein with tetratricopeptide repeats 1 | -2.07 | ***3.00*** | ***8.04*** |
| FAM69A | family with sequence similarity 69, member A | -1.07 | ***2.99*** | 1.62 |
| 17070242 | Unknown or uncharacterized | 1.09 | ***2.99*** | ***6.16*** |
| NARS | asparaginyl-tRNA synthetase | 1.20 | ***2.99*** | 1.58 |
| IFI16 | interferon, gamma-inducible protein 16 | 1.55 | ***2.94*** | 3.91 |
| EPSTI1 | epithelial stromal interaction 1 (breast) | -1.18 | ***2.93*** | ***6.69*** |
| SDK2 | sidekick cell adhesion molecule 2 | -1.27 | ***2.87*** | 1.30 |
| 17123260 | Unknown or uncharacterized | -1.03 | ***2.87*** | ***4.91*** |
| 17123268 | Unknown or uncharacterized | -1.29 | ***2.76*** | ***5.65*** |
| PNPT1 | polyribonucleotide nucleotidyltransferase 1 | 1.19 | ***2.74*** | ***6.56*** |
| OAS3 | 2'-5'-oligoadenylate synthetase 3, 100kDa | -2.06 | ***2.74*** | ***3.92*** |
| 16963218 | snRNA | 1.26 | ***2.72*** | -1.15 |
| SEC61B | Sec61 beta subunit | ***2.47*** | ***2.69*** | 2.33 |
| OAS1 | 2'-5'-oligoadenylate synthetase 1, 40/46kDa | 1.06 | ***2.69*** | ***3.52*** |
| IFI6 | interferon, alpha-inducible protein 6 | 1.02 | ***2.68*** | ***8.53*** |
| 17120106 | Unknown or uncharacterized | -1.01 | ***2.68*** | ***2.39*** |
| IL6ST | Interleukin 6 signal transducer | 1.11 | ***2.67*** | 1.89 |
| 16657279 | Unknown or uncharacterized | 1.44 | ***2.67*** | 1.23 |
| PML | promyelocytic leukemia | -1.24 | ***2.65*** | ***4.30*** |
| SDK2 | sidekick cell adhesion molecule 2 | -1.27 | ***2.64*** | 1.30 |
| KIAA0664L3 | KIAA0664-like 3 | 1.51 | ***2.63*** | ***2.23*** |
| KSR1 | kinase suppressor of ras 1 | 1.07 | ***2.60*** | 1.88 |
| RSAD2 | radical S-adenosyl methionine domain containing 2 | -1.43 | ***2.60*** | ***9.26*** |
| DDX60* | DEAD (Asp-Glu-Ala-Asp) box polypeptide 60 | -1.63 | ***2.59*** | ***7.40*** |
| CLEC2D | C-type lectin domain family 2, member D | -1.26 | ***2.58*** | ***9.81*** |
| CMPK2 | cytidine monophosphate (UMP-CMP) kinase 2, mitochondrial | -1.71 | ***2.55*** | ***5.27*** |
| PARP12 | poly (ADP-ribose) polymerase family, member 12 | -1.06 | ***2.54*** | ***3.49*** |
| BCL2L1 | BCL2-like 1 | -1.22 | ***2.54*** | ***3.95*** |
| VAV2 | vav 2 guanine nucleotide exchange factor | -1.20 | ***2.53*** | 1.91 |
| COPZ1 | coatomer protein complex, subunit zeta 1 | 1.24 | ***2.49*** | -1.00 |
| HERC6 | HECT and RLD domain containing E3 ubiquitin protein ligase | -1.43 | ***2.48*** | ***7.08*** |
| LOC730101 | uncharacterized LOC730101 | 1.19 | ***2.46*** | 1.72 |
| STAT2 | Signal transducer and activator of transcription 2 | 1.25 | ***2.44*** | ***2.43*** |
| IRF2 | interferon regulatory factor 2 | -1.12 | ***2.44*** | ***4.38*** |
| CFL1 | cofilin 1 (non-muscle) | 1.04 | ***2.42*** | 1.23 |
| HINT1 | histidine triad nucleotide binding protein 1 | 1.31 | ***2.40*** | 1.47 |
| ICOS | inducible T-cell co-stimulator | 1.73 | ***2.41*** | 5.42 |
| BCL2L1 | BCL2-like 1 | 1.44 | ***2.39*** | ***3.95*** |
| 16655261 | Unknown or uncharacterized | ***2.17*** | ***2.37*** | 2.05 |
| RN5S51 | RNA, 5S ribosomal 51 | 1.21 | ***2.37*** | 1.26 |
| ABCB10 | ATP-binding cassette, sub-family B (MDR/TAP) | 1.15 | ***2.37*** | 1.34 |
| IFI44 * | interferon-induced protein 44 | -1.70 | ***2.36*** | ***2.85*** |
| LAMP3 | lysosomal-associated membrane protein 3 | -1.37 | ***2.34*** | ***5.74*** |
| STAP1 | signal transducing adaptor family member 1 | 1.58 | ***2.34*** | ***3.47*** |
| 16907904 | Unknown or uncharacterized | 1.46 | ***2.31*** | 2.40 |
| TMCC3 | transmembrane and coiled-coil domain family 3 | 1.42 | ***2.28*** | ***2.39*** |
| ASPHD2 | aspartate beta-hydroxylase domain containing 2 | 1.20 | ***2.22*** | ***10.57*** |
| C19orf66 | chromosome 19 open reading frame 66 | 1.11 | ***2.21*** | ***3.07*** |
| PHGDH | phosphoglycerate dehydrogenase | -1.02 | ***2.18*** | ***1.81*** |
| FCRL3 | Fc receptor-like 3 | -1.12 | ***2.18*** | 1.75 |
| 16655639 | Unknown or uncharacterized | ***3.21*** | ***2.17*** | ***2.25*** |
| 17069479 | misc_RNA | 1.77 | ***2.17*** | 2.03 |
| SP100 | SP100 nuclear antigen | -1.15 | ***2.16*** | ***2.52*** |
| ZER1 | zer-1 homolog (C. elegans) | 1.01 | ***2.14*** | ***1.97*** |
| NPM1 | nucleophosmin (nucleolar phosphoprotein B23, numatrin) | 1.04 | ***2.11*** | 1.61 |
| 16655153 | Unknown or uncharacterized | -1.82 | ***2.11*** | ***2.00*** |
| SCYL3 | SCY1-like 3 (S. cerevisiae) | -1.15 | ***2.09*** | ***2.37*** |
| 16650623 | Unknown or uncharacterized | 1.21 | ***2.08*** | -1.17 |
| C4orf46 | chromosome 4 open reading frame 46 | 1.47 | ***2.08*** | ***2.02*** |
| HSH2D | hematopoietic SH2 domain containing, RAB8A | -1.24 | ***2.07*** | ***2.99*** |
| FMNL3 | formin-like 3 | 1.17 | ***2.07*** | 1.32 |
| LOC100509457 | major histoc complex, class II, DQ alpha 1 | 1.45 | ***2.07*** | 1.11 |
| PDGFRL | platelet-derived growth factor receptor-like | 1.24 | ***2.07*** | ***5.57*** |
| 16655889 | Unknown or uncharacterized | ***1.82*** | ***2.06*** | ***2.06*** |
| NEIL1 | nei endonuclease VIII-like 1 (E. coli), microRNA 631 | -1.25 | ***2.05*** | 1.29 |
| 16653841 | Unknown or uncharacterized | 1.12 | ***2,04*** | 1.34 |
| STAT1 | Signal transducer and activator of transcription 1 | -1.15 | ***2.03*** | ***1.80*** |
| ZNF584 | zinc finger protein 584 | 1.26 | ***2.03*** | 1.12 |
| P140L | SP140 nuclear body protein-like | -1.80 | ***2.02*** | ***2.62*** |
| IGHG3 | immunoglobulin heavy constant gamma 3 | 1.12 | ***2.01*** | 1.09 |
| NFAT5 | nuclear factor of activated T-cells 5 | -1.41 | ***2.00*** | ***3.88*** |
| CD274 | CD274 molecule | -1.48 | ***1.98*** | 12.84 |
| TAP1 | transporter 1, ATP-binding cassette, sub-family B | 1.10 | ***1.98*** | ***2.17*** |
| NFATC2 | nuclear factor of activated T-cells | 2.33 | ***1.97*** | 1.43 |
| CD180 | CD180 molecule | -1.74 | ***1.96*** | 1.15 |
| ARHGEF17 | Rho guanine nucleotide exchange factor (GEF) 17 | 1.06 | ***1.96*** | 1.08 |
| ENDOG | endonuclease G | 1.12 | ***1.96*** | 1.49 |
| MLLT11 | myeloid/lymphoid or mixed-lineage leukemia | 1.54 | ***1.95*** | **2.29** |
| SIPA1L1 | signal-induced proliferation-associated 1 like 1 | -1.18 | ***1.95*** | ***3.62*** |
| 17123104 | Unknown or uncharacterized | 1.39 | ***1.95*** | 2.07 |
| DNAJC6 | DnaJ (Hsp40) homolog, subfamily C, member 6 | 1.37 | ***1.94*** | 1.51 |
| MLLT11 | myeloid/lymphoid or mixed-lineage leukemia | 1.54 | ***1.94*** | ***2.29*** |
| 16655203 | Unknown or uncharacterized | 1.15 | ***1.93*** | ***3.20*** |
| RUNX3 | runt-related transcription factor 3 | -1.22 | ***1.93*** | 1.25 |
| LOC399715 | uncharacterized LOC399715 | 1.09 | ***1.93*** | ***2.92*** |
| EBF1 | early B-cell factor 1 | -1.78 | ***1.93*** | *2.28* |
| ADAR * | adenosine deaminase, RNA-specific | 1.16 | ***1.92*** | ***1.95*** |
| 16652175 | Unknown or uncharacterized | ***2.82*** | ***1.92*** | -1.04 |
| 16655849 | Unknown or uncharacterized | 1.23 | ***1,90*** | 1.06 |
| ILF2 | interleukin enhancer binding factor 2, 45kDa | 1.24 | ***1.90*** | 1.39 |
| MORN3 | MORN repeat containing 3 | 1.00 | ***1.90*** | -1.03 |
| SLAMF6 | SLAM family member 6 | -1.54 | ***1.87*** | ***2.10*** |
| 16655655 | Unknown or uncharacterized | 1.39 | ***1.86*** | 1.47 |
| PEAK1 | NKF3 kinase family member | -1.11 | ***1.86*** | ***1.85*** |
| 16989052 | misc_RNA | 1.29 | ***1.86*** | ***7.16*** |
| TXN | thioredoxin | 1.14 | ***1.86*** | ***2.93*** |
| DHX58 | DEXH (Asp-Glu-X-His) box polypeptide 58 | -1.23 | ***1.86*** | ***5.12*** |
| IFI35 | interferon-induced protein 35 | -1.11 | ***1.86*** | 2.74 |
| MX1 | Myxovirus resistance 1, ifn-inducible protein p78 | 1.10 | ***1.85*** | ***2.06*** |
| FAS | Fas (TNF receptor superfamily, member 6) | -1.12 | ***1.85*** | 1.46 |
| 16652333 | Unknown or uncharacterized | ***1.82*** | ***1.85*** | -1.23 |
| 16809913 | snoRNA | 1.70 | ***1.85*** | 1.33 |
| 16656339 | Unknown or uncharacterized | 1.60 | ***1,84*** | 1.29 |
| 16826110 | Unknown or uncharacterized | -1.10 | ***1.84*** | -1.10 |
| NCS1 | neuronal calcium sensor 1 | 1.04 | ***1.84*** | 3.15 |
| 17091271 | snoRNA | 1.11 | ***1.83*** | -1.08 |
| CD200 | CD200 molecule | -1.67 | ***1.83*** | 3.20 |
| CCR7 | Chemokine (C-C motif) receptor 7 | 1.19 | ***1.81*** | ***2.17*** |
| 16650207 | Unknown or uncharacterized | ***2.12*** | ***1.80*** | 1.62 |
| SPATS2L | spermatogenesis associated, serine-rich 2-like | 1.07 | ***1.80*** | ***2.50*** |
| 17119972 | Unknown or uncharacterized | 1.54 | ***1.80*** | 1.20 |
| 16651963 | Unknown or uncharacterized | 1.31 | ***1.78*** | 1.16 |
| 16652627 | Unknown or uncharacterized | 1.31 | ***1.78*** | 1.16 |
| 16893663 | Unknown or uncharacterized | 1.68 | ***1.78*** | 1.87 |
| C3orf26 | chromosome 3 open reading frame 26 | -1.09 | ***1.78*** | 1.69 |
| 16768265 | Unknown or uncharacterized | 1.09 | ***1.77*** | ***4.06*** |
| 17118656 | Unknown or uncharacterized | -1.62 | ***1.77*** | 1.68 |
| ARF3 | ADP-ribosylation factor 3 | -1.15 | ***1.75*** | -1.05 |
| ARHGEF3 | Rho guanine nucleotide exchange factor (GEF) 3 | 1.40 | ***1.75*** | ***1.97*** |
| 16654751 | Unknown or uncharacterized | 1.21 | ***1.74*** | 1.17 |
| SIDT1 | SID1 transmembrane family, member 1 | 1.24 | ***1.74*** | ***2.45*** |
| 16880678 | Unknown or uncharacterized | 1.65 | ***1.73*** | 1.08 |
| 16653083 | Unknown or uncharacterized | 1.19 | ***1.72*** | 1.22 |
| IFIT5 | interferon-induced protein with tetratricopeptide repeats 5 | -1.29 | ***1.72*** | ***4.50*** |
| TAP2 | transporter 2, ATP-binding cassette, sub-family B | -1.12 | ***1.72*** | 1.52 |
| MASTL | microtubule associated serine/threonine kinase-like | -1.10 | ***1.72*** | ***3.53*** |
| MIR1-1 | microRNA 1-1 | 1.43 | ***1.72*** | 1.38 |
| 16709068 | snRNA | 1.35 | ***1.71*** | 1.32 |
| IL13 | Interleukin 13 | 1.42 | ***1.71*** | 1.22 |
| PRPF8 | PRP8 pre-mRNA processing factor 8 homolog (S. cerevisiae) | 1.34 | ***1.71*** | 1.08 |
| SULT1B1 | sulfotransferase family, cytosolic, 1B, member 1 | -1.21 | ***-1.71*** | ***-1.73*** |
| KRT23 | keratin 23 (histone deacetylase inducible) | -1.25 | ***-1.74*** | -1.66 |
| 17064282 | Unknown or uncharacterized | -1.32 | ***-1.74*** | -1.23 |
| MIR221 | microRNA 221 | ***-2.53*** | ***-1.75*** | 3.53 |
| ANTXR2 | anthrax toxin receptor 2 | -1.24 | ***-1.79*** | -1.01 |
| F5 | coagulation factor V (proaccelerin, labile factor) | -1.61 | ***-1.80*** | 1.08 |
| B4GALT1 | UDP-Gal:betaGlcNAc beta1,4-galactosyltransferase | 1.05 | ***-1.80*** | -1.57 |
| 16891553 | misc_RNA | -1.09 | ***-1.80*** | 1.13 |
| ANXA1 | annexin A1 | -1.20 | ***-1.81*** | 1.12 |
| 16650899 | Unknown or uncharacterized | ***-1.78*** | ***-1.84*** | -1.84 |
| DHRS9 | dehydrogenase/reductase (SDR family) member 9 | -1.21 | ***-1.85*** | 1.65 |
| 16654315 | Unknown or uncharacterized | ***-1.80*** | ***-1.86*** | -1.23 |
| 16650871 | Unknown or uncharacterized | -1.09 | ***-1.88*** | -1.63 |
| CHMP1B | charged multivesicular body protein 1B | 1.56 | ***-1.88*** | ***-1.71*** |
| MIR548AN | microRNA 548an | -1.15 | ***-1.88*** | -1.42 |
| LOC100509635 | uncharacterized LOC100509635 | -1.20 | ***-1.89*** | 1.47 |
| CLEC6A | C-type lectin domain family 6, member A | -1.29 | ***-1.89*** | 1.66 |
| ETS2 | v-ets erythroblastosis virus E26 oncogene homolog 2 (avian) | -1.35 | ***-1.94*** | 1.26 |
| FAM117B | family with sequence similarity 117, member B | -1.62 | ***-1.96*** | ***-2.08*** |
| PIGA | phosphatidylinositol glycan anchor biosynthesis, class A | -1.12 | ***-1.96*** | -1.26 |
| 16741265 | miRNA | -1.27 | ***-1.98*** | -1.73 |
| MAPKAPK3 | mitogen-activated prot kinase-actv prot kinase 3 | -1.38 | ***-1.98*** | -1.71 |
| CEBPB | CCAAT/enhancer binding protein (C/EBP), beta | -1.47 | ***-2.02*** | -1.17 |
| CEBPB | CCAAT/enhancer binding protein (C/EBP), beta | -1.47 | ***-2.02*** | -1.17 |
| MAPK6 | mitogen-activated protein kinase 6 | -1.33 | ***-2.04*** | -1.21 |
| 17120680 | Unknown or uncharacterized | -1.20 | ***-2.09*** | 1.31 |
| SOX4 | SRY (sex determining region Y)-box 4 | 1.92 | ***-2.10*** | -1.97 |
| LRRK2 | leucine-rich repeat kinase 2 | 1.01 | ***-2.14*** | -1.34 |
| OTUD1 | OTU domain containing 1 | -1.15 | ***-2.14*** | 1.09 |
| AREG | amphiregulin | 1.99 | ***-2.14*** | 2.14 |
| CLEC4C | C-type lectin domain family 4, member C | 1.30 | ***-2.20*** | -2.81 |
| MYADM | myeloid-associated differentiation marker | -1.02 | ***-2.21*** | ***-3.11*** |
| AIF1 | allograft inflammatory factor 1 | -1.63 | ***-2.22*** | ***-3.16*** |
| TSPO | translocator protein (18kDa) | -1.39 | ***-2.25*** | -1.41 |
| IRG1 | immunoresponsive 1 homolog (mouse) | -1.16 | ***-2.31*** | -1.11 |
| VAV3 | vav 3 guanine nucleotide exchange factor | 1.33 | ***-2.32*** | -1.86 |
| LOC338758 | uncharacterized LOC338758 | -1.53 | ***-2.38*** | -1.07 |
| PHLDA1 | pleckstrin homology-like domain, family A | ***-1.77*** | ***-2.38*** | -1.15 |
| LOC100507600 | uncharacterized LOC100507600 | ***-2.80*** | ***-1.99*** | -1.55 |
| CLEC12A | C-type lectin domain family 12, member A | ***-1.80*** | ***-2.49*** | 1.24 |
| JMY | junction mediating and regulatory protein, p53 | -1.21 | ***-2.53*** | -2.10 |
| ANKRD28 | ankyrin repeat domain 28 | -1.48 | ***-2.56*** | -1.32 |
| CCL20 | Chemokine (C-C motif) ligand 20 | -1.41 | ***-2.59*** | 1.25 |
| 16916483 | snRNA | -1.01 | ***-2.59*** | -1.61 |
| ATP2B1 | ATPase, Ca++ transporting, plasma membrane 1 | ***-1.93*** | ***-2.64*** | -1.31 |
| EEPD1 | endonuclease/exonuclease/phosphatase family domain containing 1 | 1.02 | ***-2.66*** | -2.27 |
| PELI1 | pellino E3 ubiquitin protein ligase 1 | -2.03 | ***-2.70*** | -1.46 |
| KCTD12 | potassium channel tetramerisation domain containing 12 | -1.69 | ***-2.71*** | -1.95 |
| RRP12 | ribosomal RNA processing 12 homolog (S. cerevisiae) | -1.86 | ***-2.74*** | -2.00 |
| SMPDL3A | sphingomyelin phosphodiesterase, acid-like 3A | ***-2.85*** | ***-2.74*** | -1.49 |
| 16701951 | snRNA | -1.71 | ***-2.83*** | ***4.46*** |
| NAAA | N-acylethanolamine acid amidase | -1.26 | ***-2.89*** | -1.43 |
| SGMS2 | sphingomyelin synthase 2 | 1.03 | ***-2.99*** | -1.31 |
| TRMT5 | tRNA methyltransferase 5 homolog (S. cerevisiae) | -1.25 | ***-3.19*** | 1.27 |
| EREG | epiregulin | -1.25 | ***-3.36*** | -1.07 |
| CPVL | carboxypeptidase, vitellogenic-like | -2.67 | ***-3.36*** | -2.24 |
| TNFSF14 | tumor necrosis factor (ligand) superfamily, 14 | -2.07 | ***-3.42*** | -1.24 |
| MMP10 | matrix metallopeptidase 10 (stromelysin 2) | -2.42 | ***-3.49*** | ***4.30*** |
| 16837087 | Unknown or uncharacterized | -2.37 | ***-3.62*** | -2.11 |
| EDN1 | endothelin 1 | -1.48 | ***-3.63*** | 1.41 |
| LIPN | lipase, family member N | -1.70 | ***-3.69*** | -1.62 |
| C19orf38 | chromosome 19 open reading frame 38 | ***-2.11*** | ***-3.81*** | -2.23 |
| RGCC | regulator of cell cycle | ***-2.36*** | ***-3.84*** | -2.41 |
| MAFB | v-maf musculoaponeurotic fibrosarcoma oncogene homolog B (avian) | -2.75 | ***-3.88*** | -2.84 |
| BST1 | bone marrow stromal cell antigen 1 | -1.96 | ***-4.02*** | -2.41 |
| CCRN4L | CCR4 carbon catabolite repression 4-like (S. cerevisiae) | -3.11 | ***-4.36*** | -2.19 |
| GUK1 | guanylate kinase 1 | 1.51 | ***-4.38*** | -1.22 |
| C5AR1 | Complement component 5a receptor 1 | -2.70 | ***-4.41*** | -2.45 |
| CXCL3 | Chemokine (C-X-C motif) ligand 3 | -2.44 | ***-4.82*** | 1.06 |
| EIF3F | eukaryotic translation initiation factor 3, subunit F | 1.11 | ***-5.08*** | 1.10 |
| PLBD1 | phospholipase B domain containing 1 | -3.36 | ***-7.41*** | -3.38 |
| VCAN | versican | -2.90 | ***-8.50*** | -6.44 |

**Table S1.** Human pDCs were left unstimulated or stimulated with A. fumigatus hyphae for 2 hr (Asp 2h), A. fumigatus hyphae for 4 hr (Asp 4h), or with CpG for 4 hr and then analyzed for gene expression by microarray as in *Methods*. The complete set of 250 genes that had significant changes in the Asp 4h group is shown along with the other stimulated groups. Data are expressed as fold change compared to unstimulated pDCs and represent the mean among three donors, except for CpG group where the data represent the mean between 2 donors. Significant (p<0.05) changes are shown in bold. Note that some genes were already included in Tables 1 – 4.
